# Supplementary material for: Polyketide Derivatives, Guhypoxylonols A–D from a Mangrove Endophytic Fungus Aspergillus sp. GXNU-Y45 That Inhibit Nitric Oxide Production
Source: Mar Drugs. 2021 Dec 21;20(1):5. doi: 10.3390/md20010005 (PMC8778885; doi:10.3390/md20010005)
Supplement: Supplementary file 1 [file marinedrugs-20-00005-s001.zip › marinedrugs-1494557-supplementary.pdf]

SUPPLEMENTARY MATERIAL

Polyketide Derivatives, guhypoxylonols A-D from a mangrove endophytic fungus *Aspergillus* sp. GXNU-Y45 that inhibit nitric oxide production

Xiaoya Qin[1], Jiguo Huang[2], Dexiong Zhou[1], Wenxiu Zhang[1], Yanjun Zhang[3], Jun Li[1], Ruiyun Yang[1*], Xishan Huang[1*]

[1] State Key Laboratory for Chemistry and Molecular Engineering of Medicinal Resources, Collaborative Innovation Center for Guangxi Ethnic Medicine, College of Chemistry and Pharmaceutical Sciences, Guangxi Normal University, Guilin, P. R. China;

[2] Guangdong Industry Polytechnic, Guangzhou, P. R. China;

[3] Guangxi Key Laboratory of Green Chemical Materials and Safety Technology, Beibu Gulf University, Qinzhou, P. R. China;

[*] Correspondence: yang_rui_yun@163.com; huangxishan13@foxmail.com; Tel.: +86-773-2120958

Abstract: Four undescribed compounds, guhypoxylonols A (**1**), B (**2**), C (**3**), and D (**4**), were isolated from the mangrove endophytic fungus *Aspergillus* sp. GXNU-Y45, together with seven previously reported metabolites. The structures of **1-4** were elucidated based on analysis of HRESIMS data, NMR spectroscopic data. The absolute configurations of the stereogenic carbons in **1-3** were established through a combination of spectroscopic data and electronic circular dichroism (ECD). Compounds **1-11** were evaluated for their anti-inflammatory activity. Compounds **1**, **3**, **4**, and **6** showed an inhibitory activity against the production of nitric oxide (NO), with the IC_{50} values of 14.42 ± 0.11, 18.03 ± 0.14, 16.66 ± 0.21, and 21.05 ± 0.13 μM, respectively.

Keywords: *Aspergillus* sp.; mangrove endophytic fungus; guhypoxylonols A-D; anti-inflammatory

List of supporting information

Figure S1. ^{1}H NMR (600 MHz, DMSO-d_6) spectrum of compound **1**
Figure S2. ^{13}C NMR (150 MHz, DMSO-d_6) spectrum of compound **1**
Figure S3. ^{1}H-^{1}H COSY (DMSO-d_6) spectrum of compound **1**
Figure S4. HMQC (DMSO-d_6) spectrum of compound **1**
Figure S5. HMBC (DMSO-d_6) spectrum of compound **1**
Figure S6. NOESY (DMSO-d_6) spectrum of compound **1**
Figure S7. HR-ESI-MS spectrum of compound **1**
Figure S8. ^{1}H NMR (600 MHz, DMSO-d_6) spectrum of compound **2**
Figure S9. ^{13}C NMR (150 MHz, DMSO-d_6) spectrum of compound **2**
Figure S10 ^{1}H-^{1}H COSY (DMSO-d_6) spectrum of compound **2**
Figure S11. HMQC (DMSO-d_6) spectrum of compound **2**
Figure S12. HMBC (DMSO-d_6) spectrum of compound **2**
Figure S13. NOESY (DMSO-d_6) spectrum of compound **2**
Figure S14. HR-ESI-MS spectrum of compound **2**
Figure S15. ^{1}H NMR (400 MHz, methanol-d_4) spectrum of compound **3**
Figure S16. ^{13}C NMR (100 MHz, methanol-d_4) spectrum of compound **3**
Figure S17. ^{1}H-^{1}H COSY (methanol-d_4) spectrum of compound **3**
Figure S18. HMQC (methanol-d_4) spectrum of compound **3**
Figure S19. HMBC (methanol-d_4) spectrum of compound **3**
Figure S20. NOESY (methanol-d_4) spectrum of compound **3**
Figure S21. HR-ESI-MS spectrum of compound **3**
Figure S22. ^{1}H NMR (400 MHz, methanol-d_4) spectrum of compound **4**
Figure S23. ^{13}C NMR (100 MHz, methanol-d_4) spectrum of compound **4**
Figure S24. HMQC (methanol-d_4) spectrum of compound **4**
Figure S25. HMBC (methanol-d_4) spectrum of compound **4**
Figure S26. HR-ESI-MS spectrum of compound **4**
Figure S27. ^{1}H NMR (600 MHz, DMSO-d_6) spectrum of compound **5**
Figure S28. ^{13}C NMR (150 MHz, DMSO-d_6) spectrum of compound **5**
Figure S29. ^{1}H NMR (600 MHz, DMSO-d_6) spectrum of compound **6**
Figure S30. ^{13}C NMR (150 MHz, DMSO-d_6) spectrum of compound **6**
Figure S31. ^{1}H NMR (600 MHz, DMSO-d_6) spectrum of compound **7**
Figure S32. ^{13}C NMR (150 MHz, DMSO-d_6) spectrum of compound **7**
Figure S33. ^{1}H NMR (600 MHz, DMSO-d_6) spectrum of compound **8**
Figure S34. ^{13}C NMR (150 MHz, DMSO-d_6) spectrum of compound **8**
Figure S35. ^{1}H NMR (600 MHz, DMSO-d_6) spectrum of compound **9**
Figure S36. ^{13}C NMR (150 MHz, DMSO-d_6) spectrum of compound **9**
Figure S37. ^{1}H NMR (600 MHz, DMSO-d_6) spectrum of compound **10**
Figure S38. ^{13}C NMR (150 MHz, DMSO-d_6) spectrum of compound **10**
Figure S39. ^{1}H NMR (600 MHz, DMSO-d_6) spectrum of compound **11**
Figure S40. ^{13}C NMR (150 MHz, DMSO-d_6) spectrum of compound **11**
Figure S41. ECD calculation of **1**

Table S1. Energy minimized coordinates of **1-1** model at the basis set def-SV(P) for all atoms(Å).

Table S2. Energy minimized coordinates of **1-2** model at the basis set def-SV(P) for all atoms(Å). **Figure S42**. ECD calculation of **2**

Table S3. Energy minimized coordinates of **2-1** model at the basis set def-SV(P) for all atoms(Å).

Table S4. Energy minimized coordinates of **2-2** model at the basis set def-SV(P) for all atoms(Å).

Table 5S. Energy minimized coordinates of **2-3** model at the basis set def-SV(P) for all atoms(Å).

Table S6. Energy minimized coordinates of **2-4** model at the basis set def-SV(P) for all atoms(Å).
Table S7. Energy minimized coordinates of **2-5** model at the basis set def-SV(P) for all atoms(Å). **Table 8.** Energy minimized coordinates of **2-6** model at the basis set def-SV(P) for all atoms(Å).
Figure S43. ECD calculation of **3**
Table S9. Energy minimized coordinates of **3-1** model at the basis set def-SV(P) for all atoms(Å).
Table S10. Energy minimized coordinates of **3-2** model at the basis set def-SV(P) for all atoms(Å).

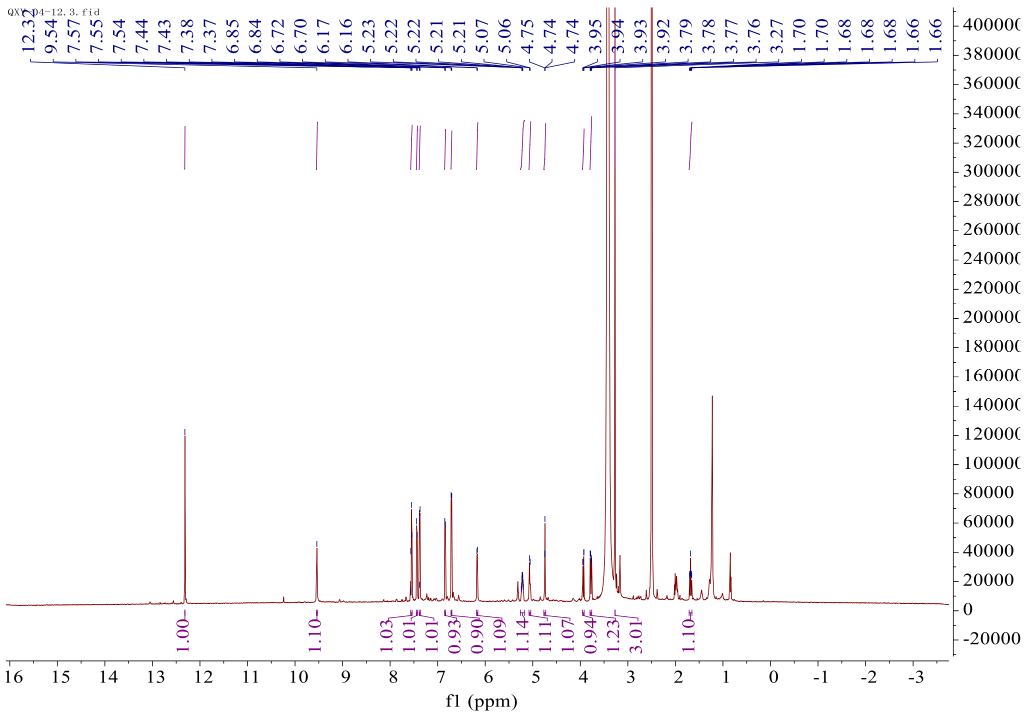

Figure S1. ^{1}H NMR (600 MHz, DMSO-d_6) spectrum of compound 1

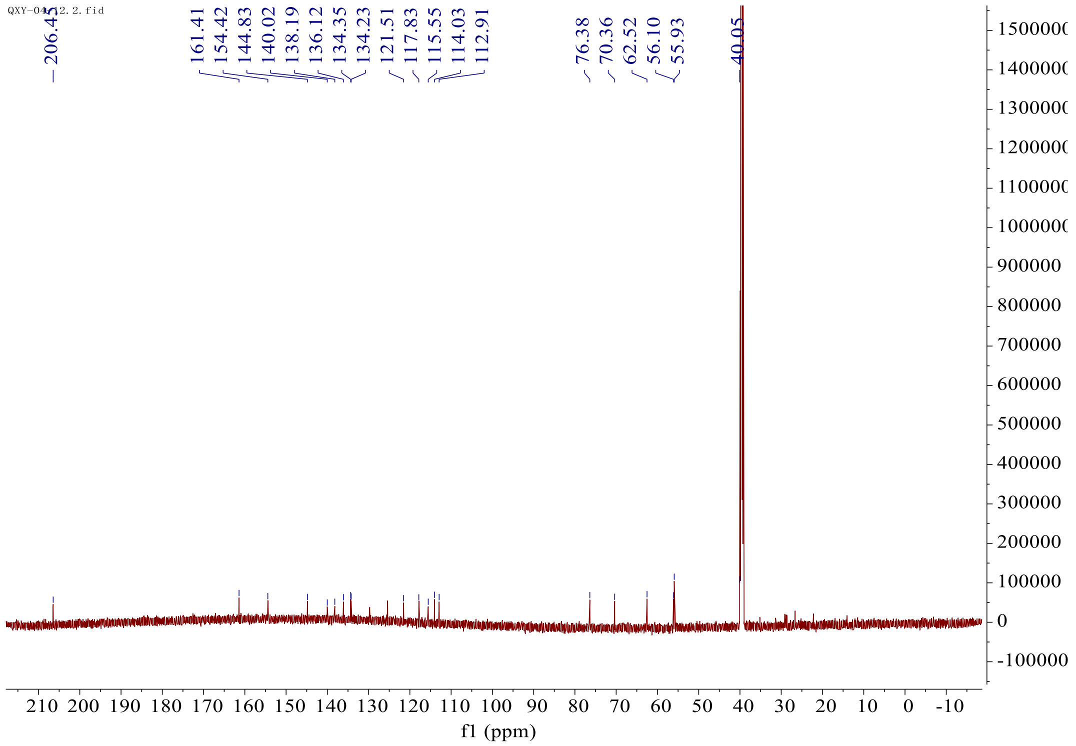

Figure S2. ^{13}C NMR (150 MHz, DMSO-d_6) spectrum of compound 1

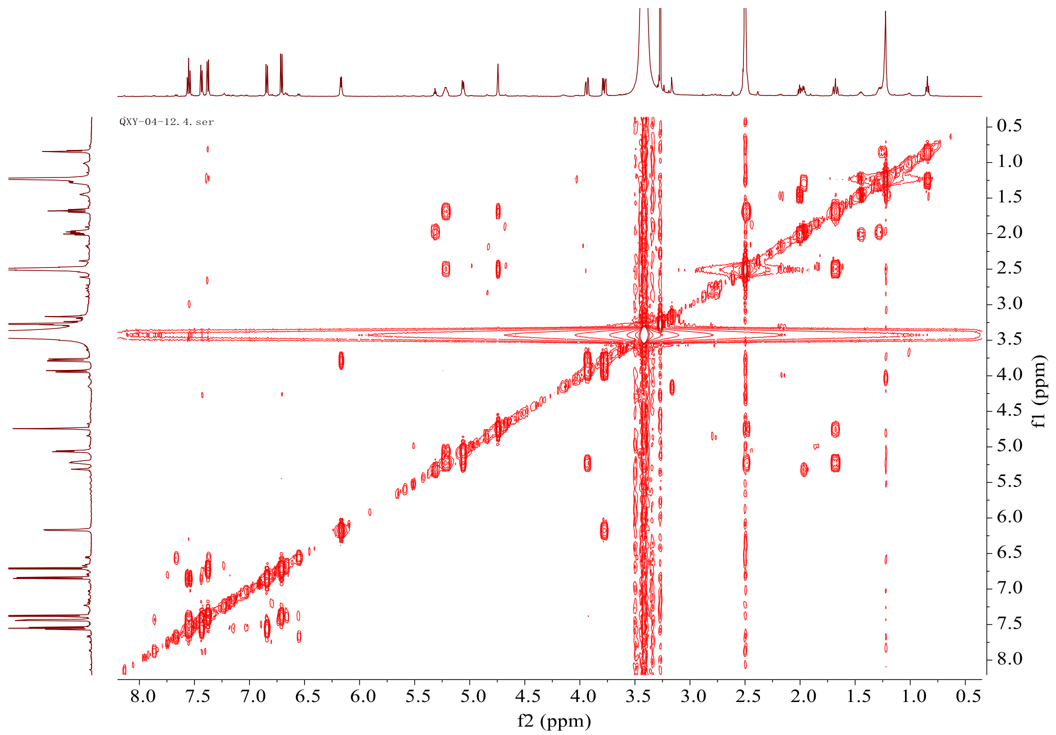

Figure S3. ${}^{1}H$-${}^{1}H$ COSY (DMSO-d_6) spectrum of compound **1**

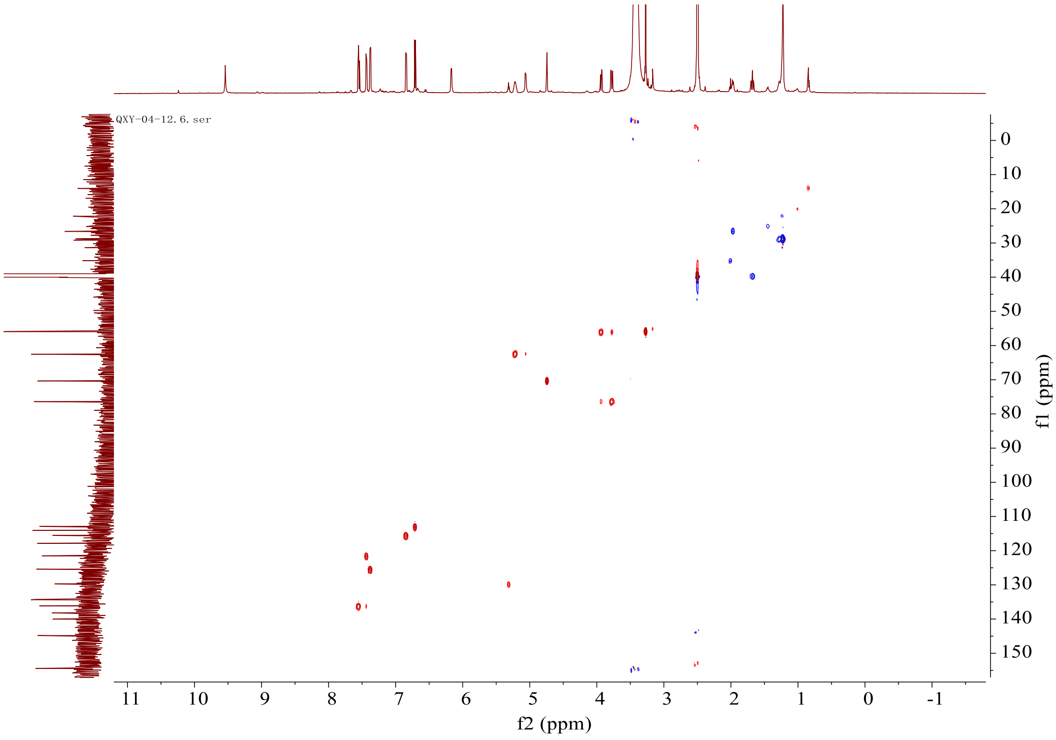

Figure S4. HMQC (DMSO-d_6) spectrum of compound **1**

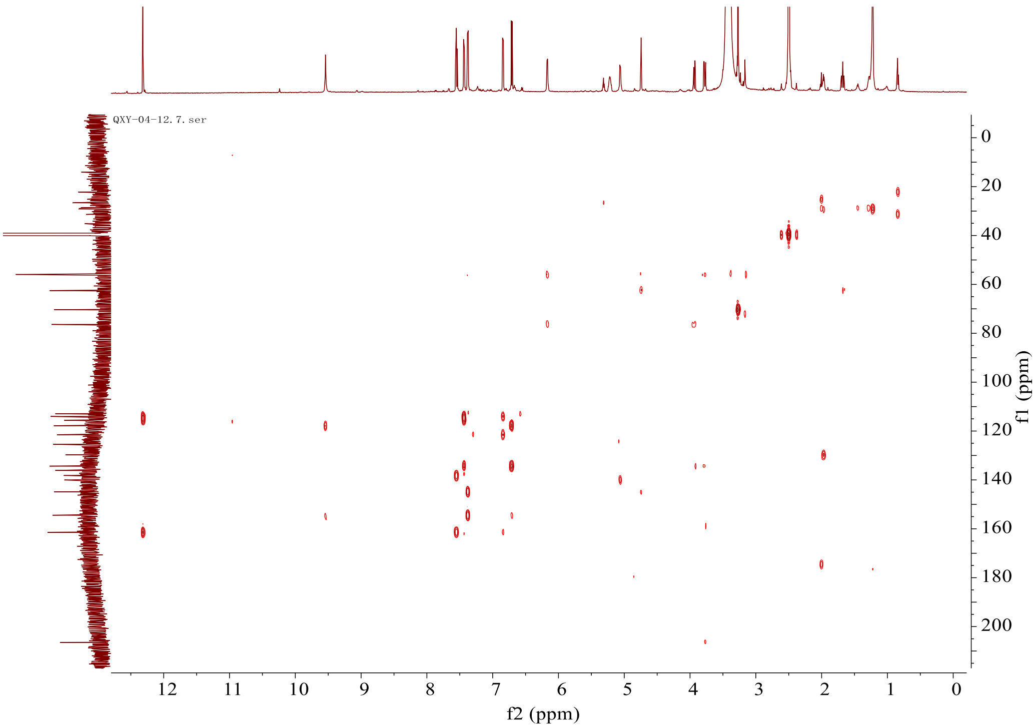

Figure S5. HMBC (DMSO-d_6) spectrum of compound **1**

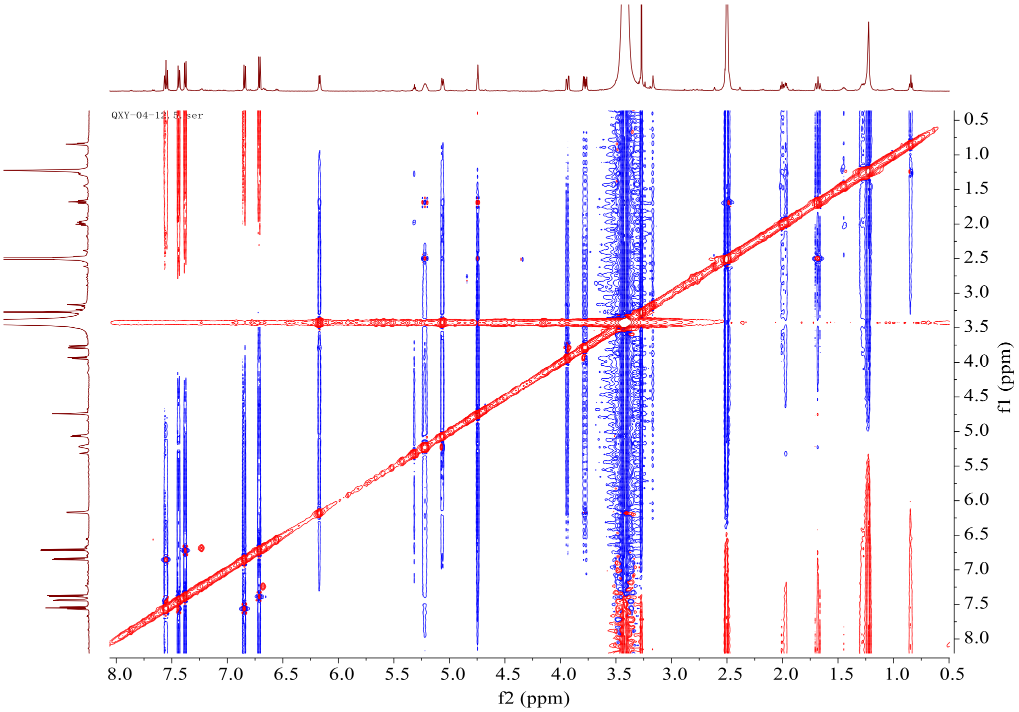

Figure S6. NOESY (DMSO-d_6) spectrum of compound **1**

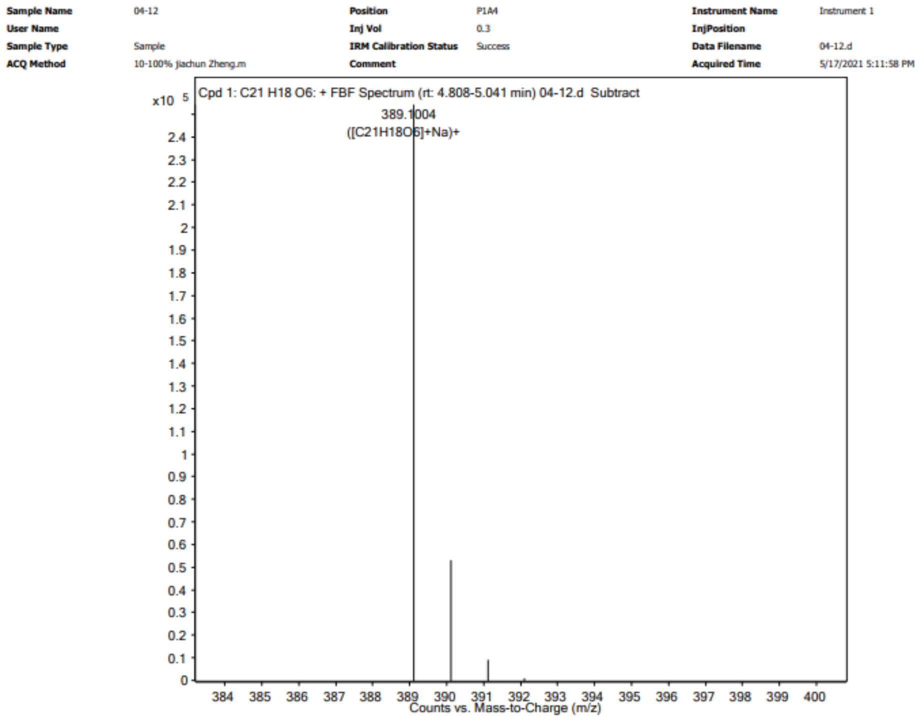

Figure S7. HR-ESI-MS spectrum of compound **1**

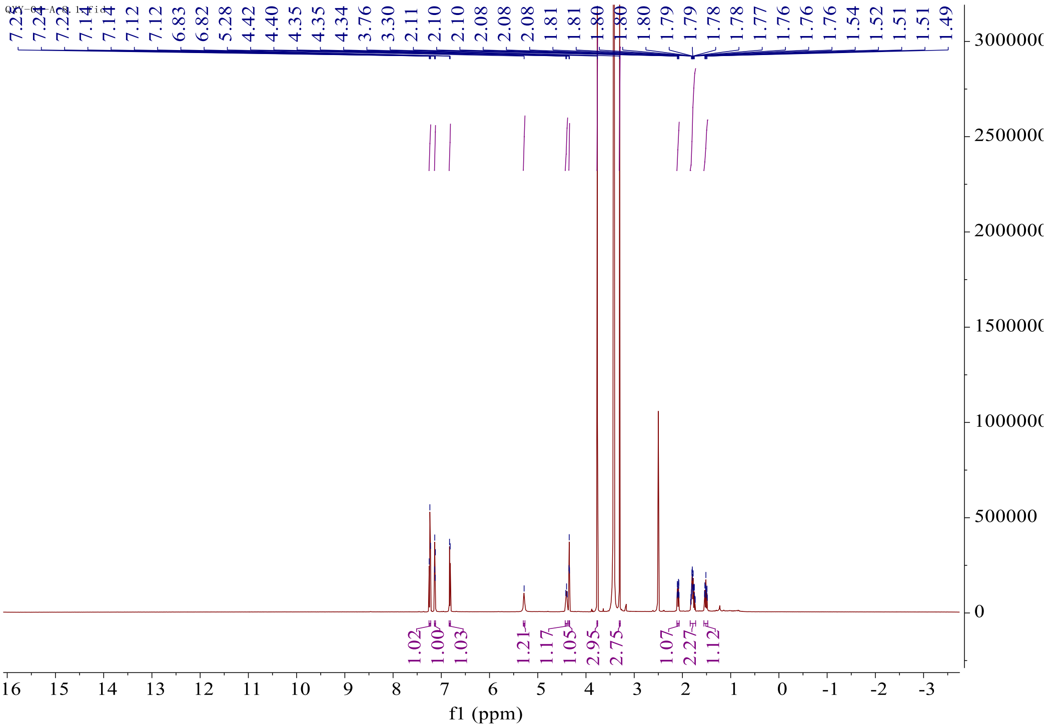

Figure S8. ^{1}H NMR (600 MHz, DMSO-d_6) spectrum of compound **2**

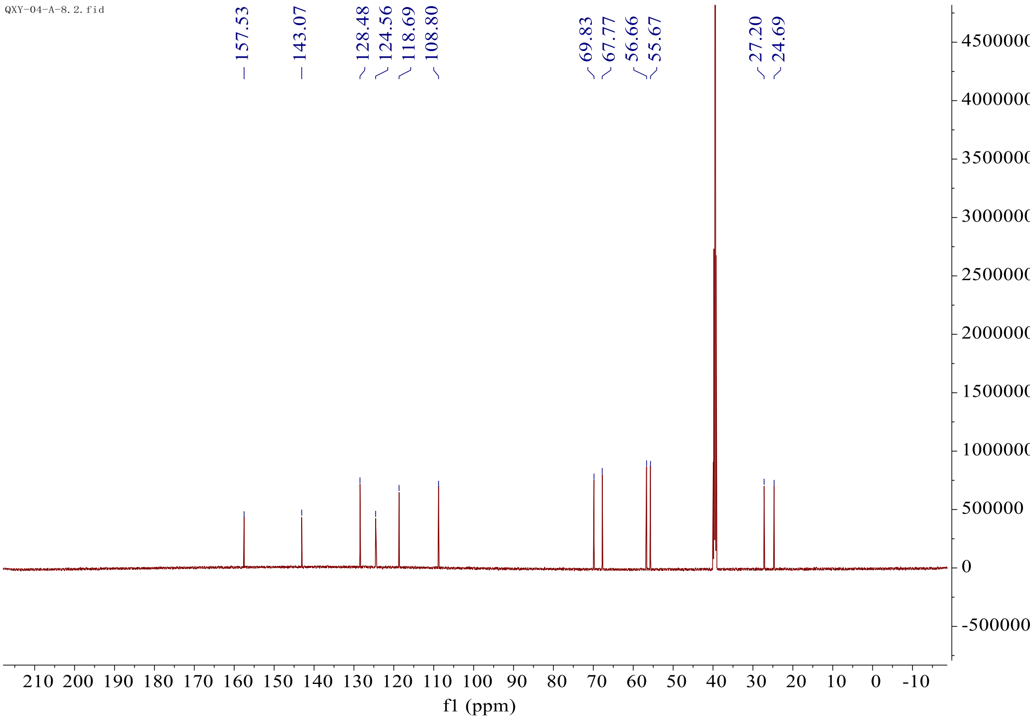

Figure S9. ^{13}C NMR (150 MHz, DMSO-d_6) spectrum of compound **2**

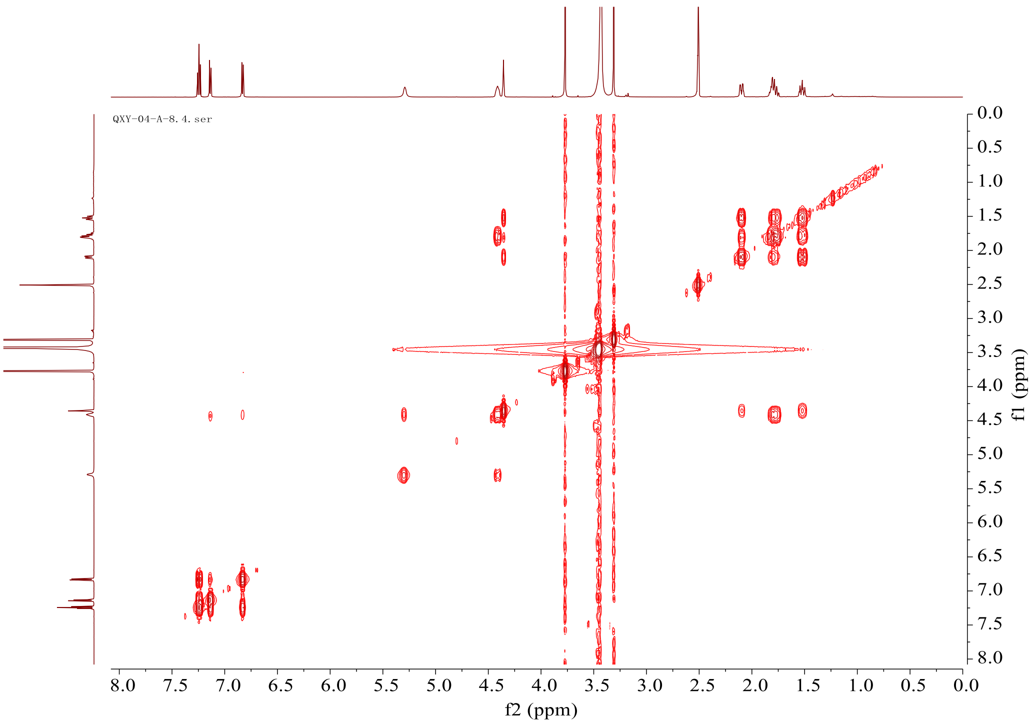

Figure S10. ^{1}H-^{1}H COSY (DMSO-d_6) spectrum of compound **2**

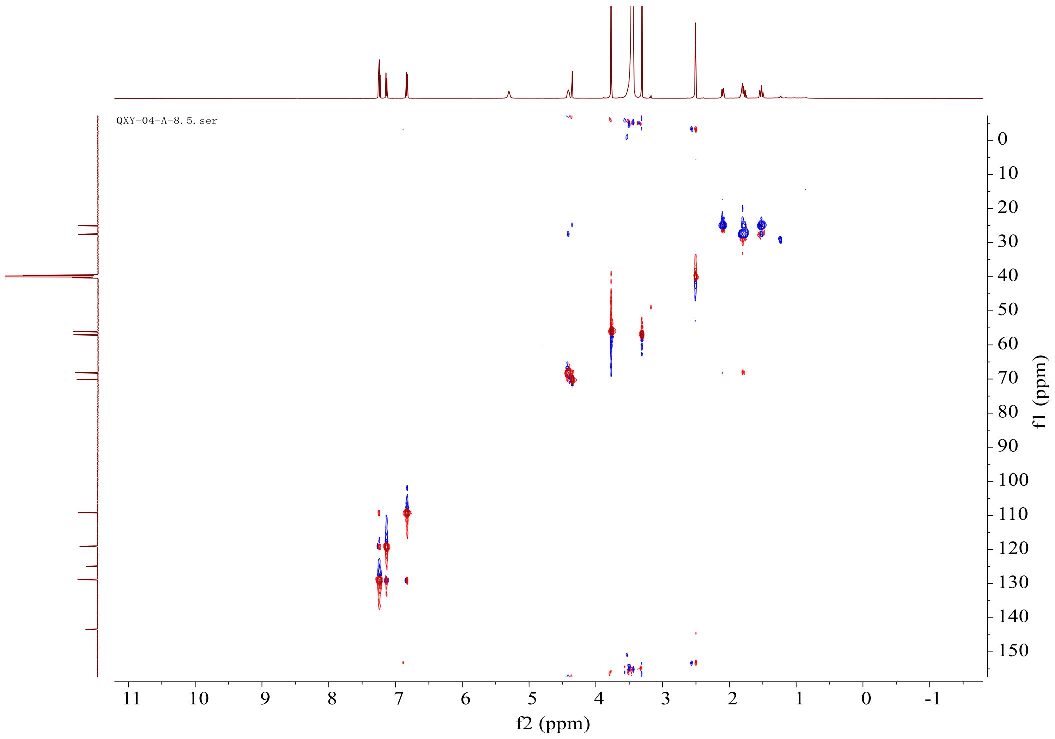

Figure S11. HMQC (DMSO-d_6) spectrum of compound **2**

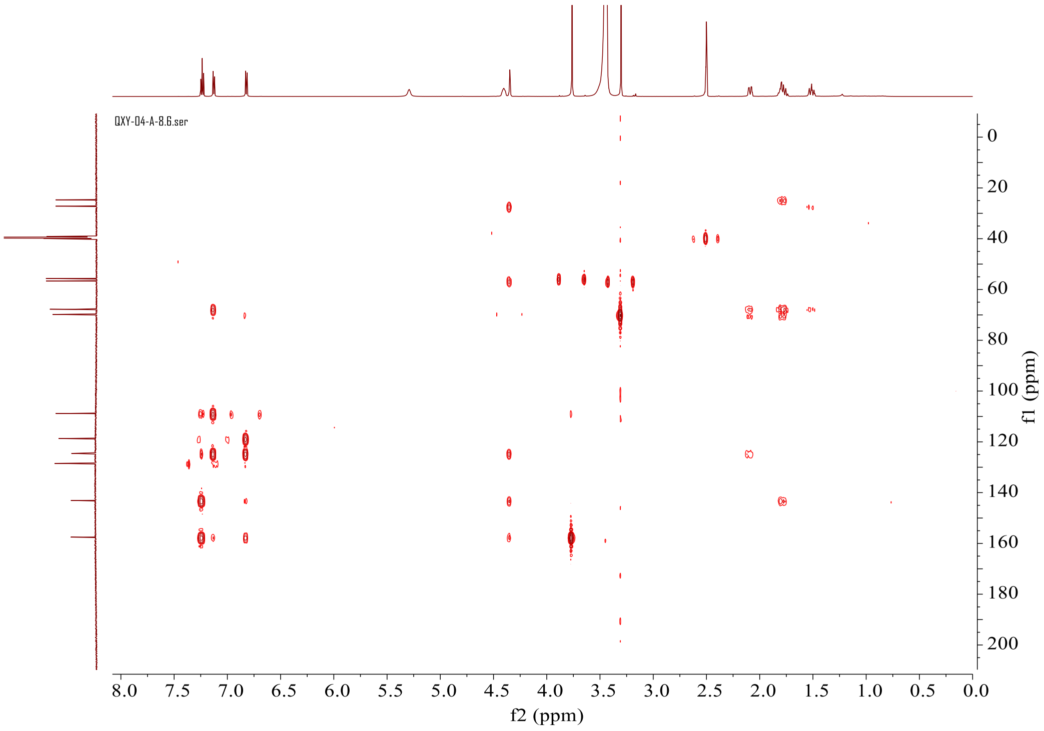

Figure S12. HMBC (DMSO-d_6) spectrum of compound **2**

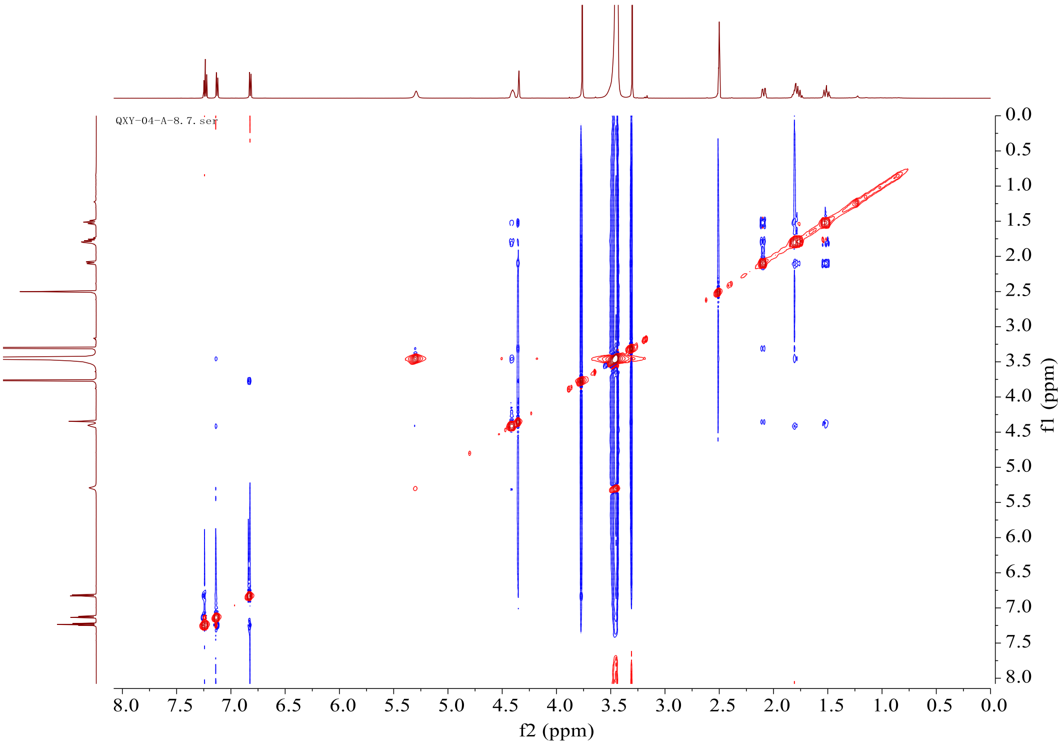

Figure S13. NOESY (DMSO-d_6) spectrum of compound **2**

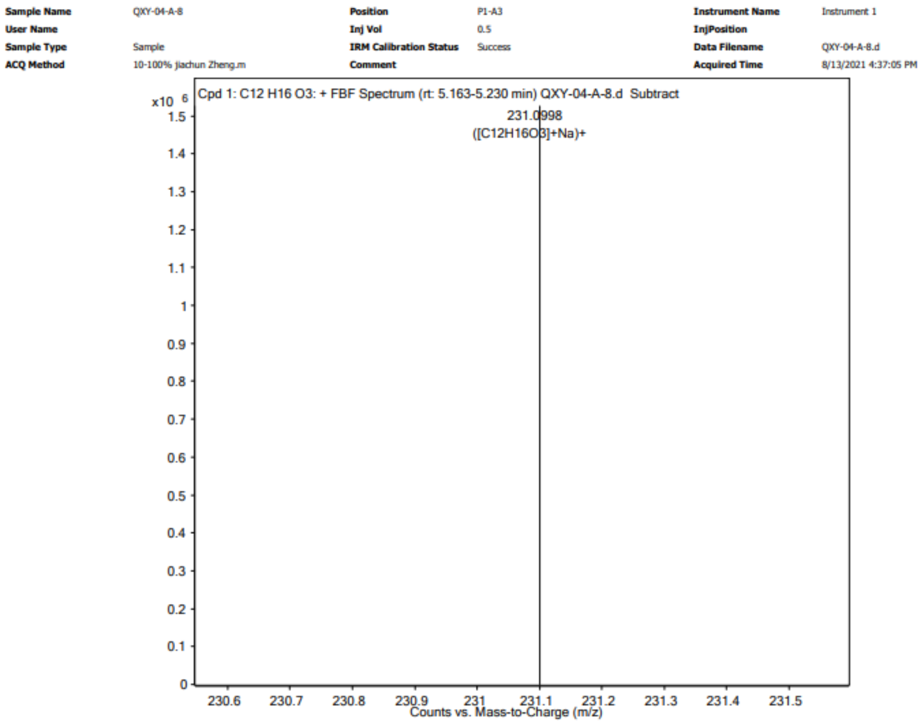

Figure S14. HR-ESI-MS spectrum of compound **2**

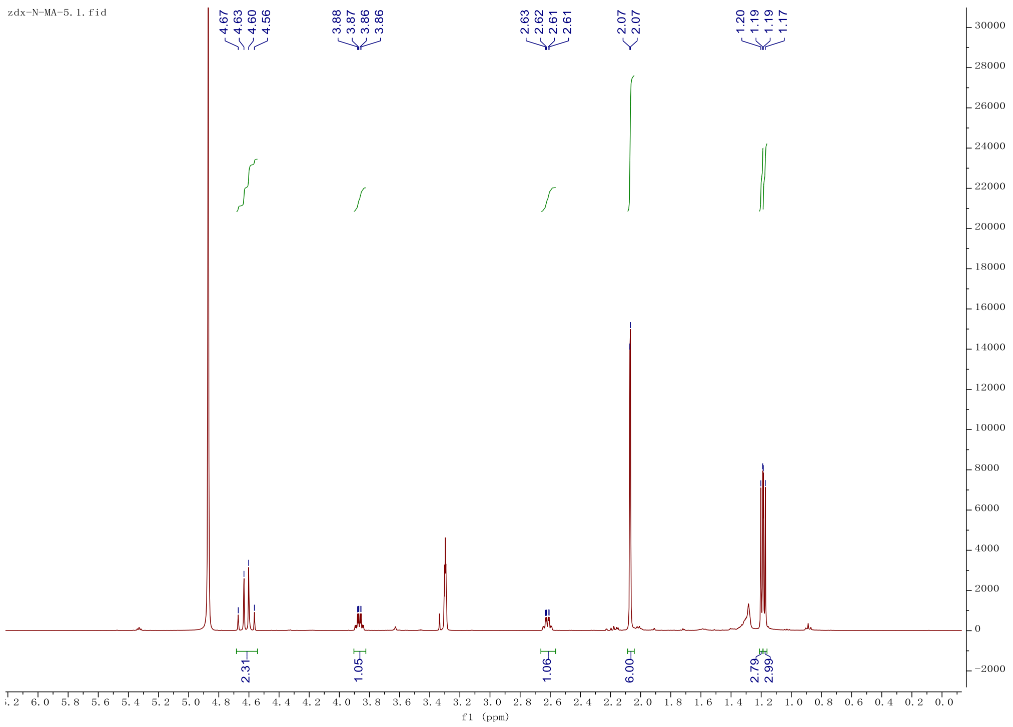

Figure S15. ^{1}H NMR (400 MHz, methanol-d_4) spectrum of compound **3**

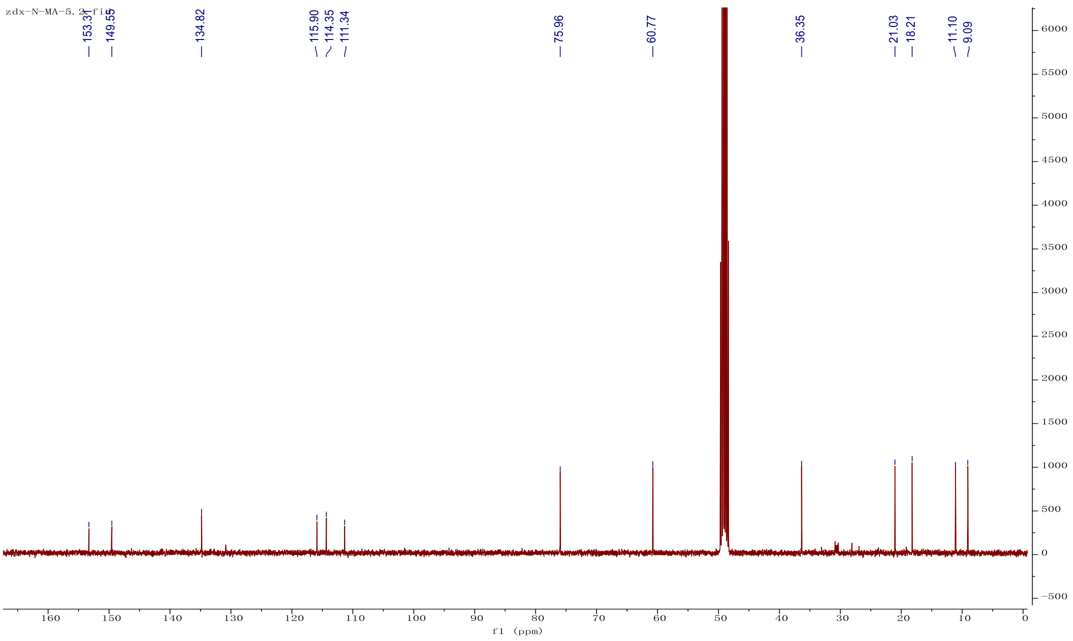

Figure S16. ^{13}C NMR (100 MHz, methanol-d_4) spectrum of compound **3**

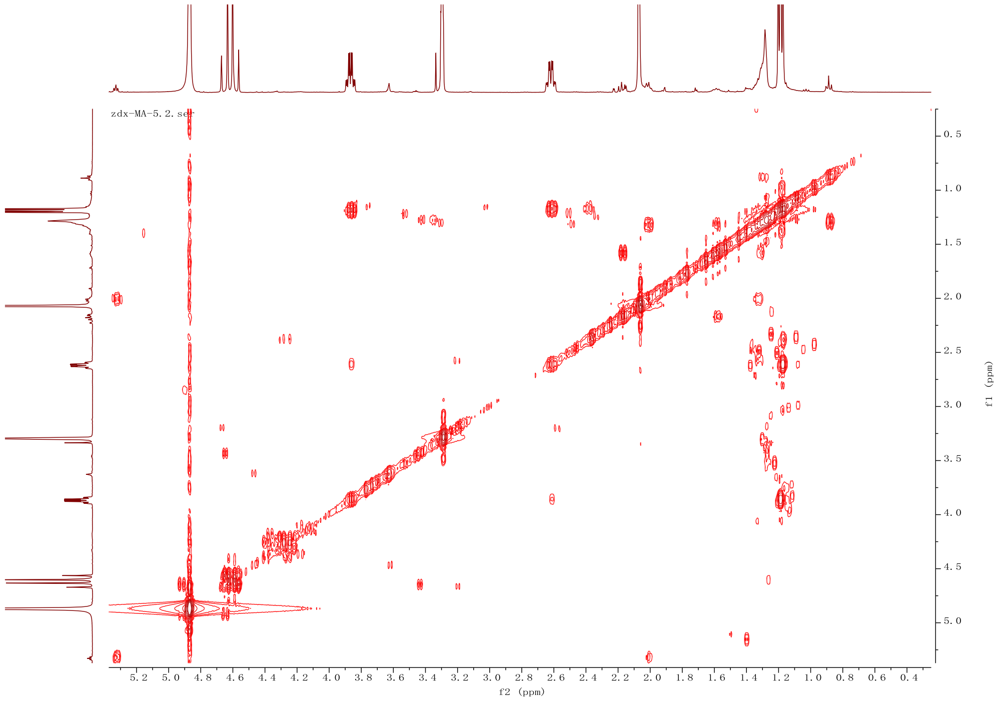

Figure S17. ^{1}H-^{1}H COSY (methanol-d_4) spectrum of compound **3**

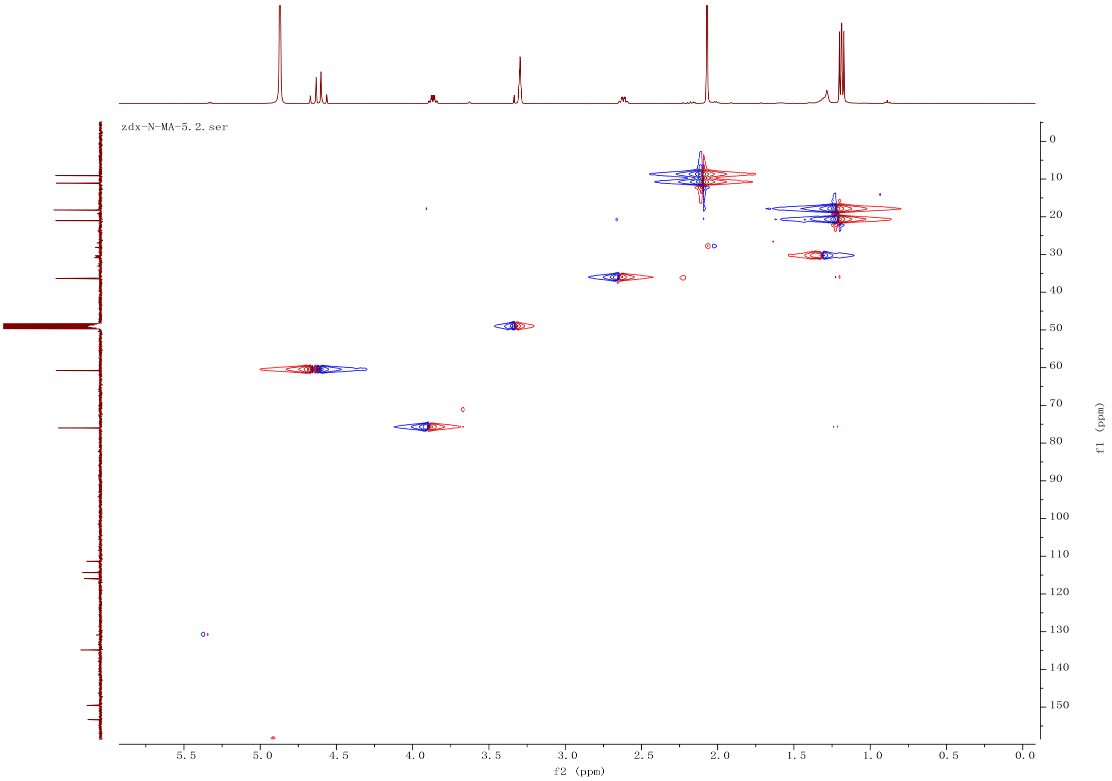

Figure S18. HMQC (methanol-d_4) spectrum of compound **3**

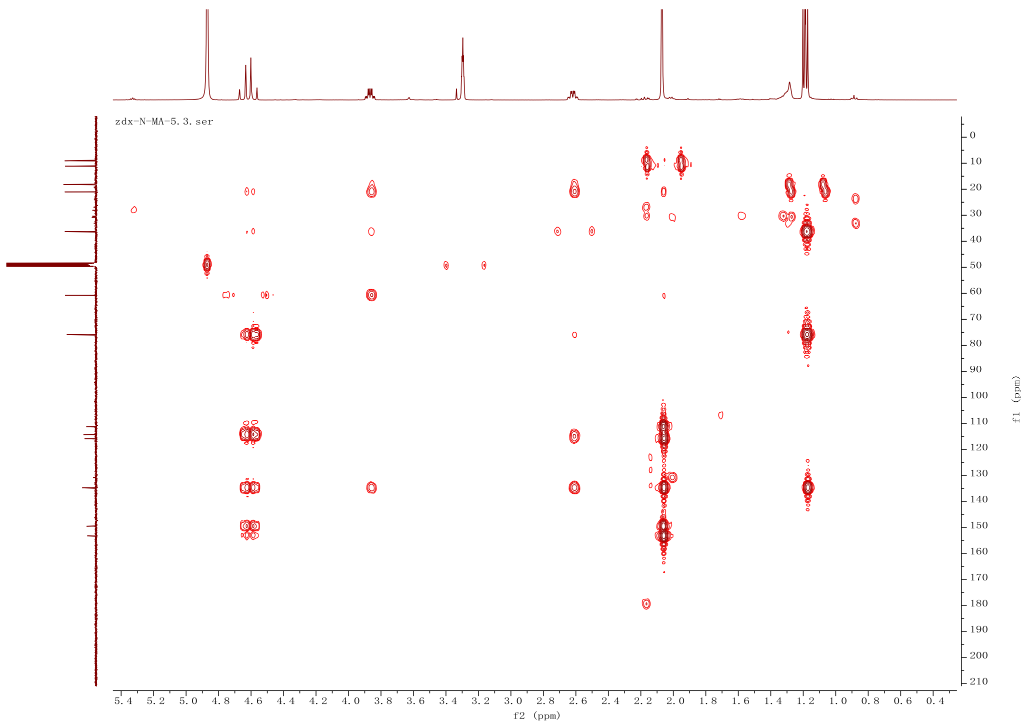

Figure S19. HMBC (methanol-d_4) spectrum of compound **3**

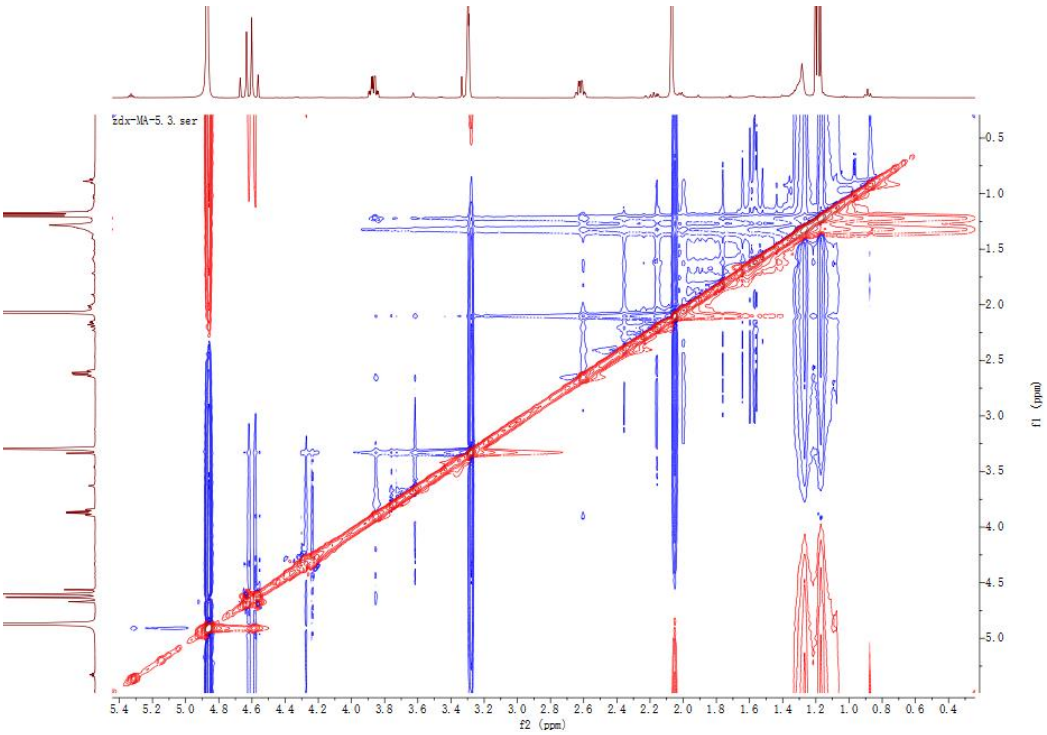

Figure S20. NOESY (methanol-d_4) spectrum of compound **3**

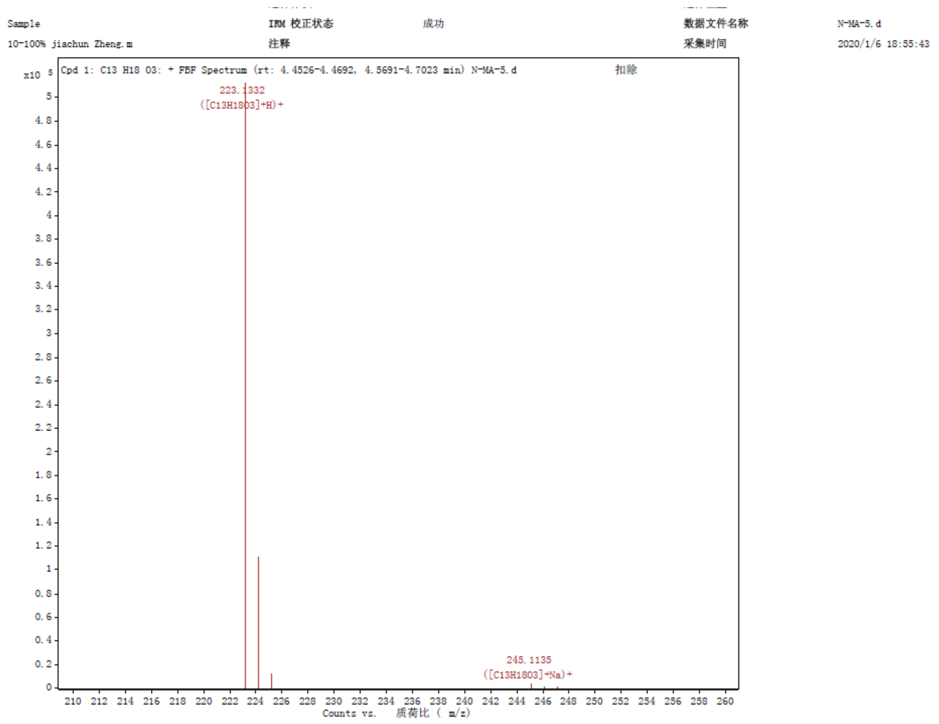

Figure S21. HR-ESI-MS spectrum of compound 3

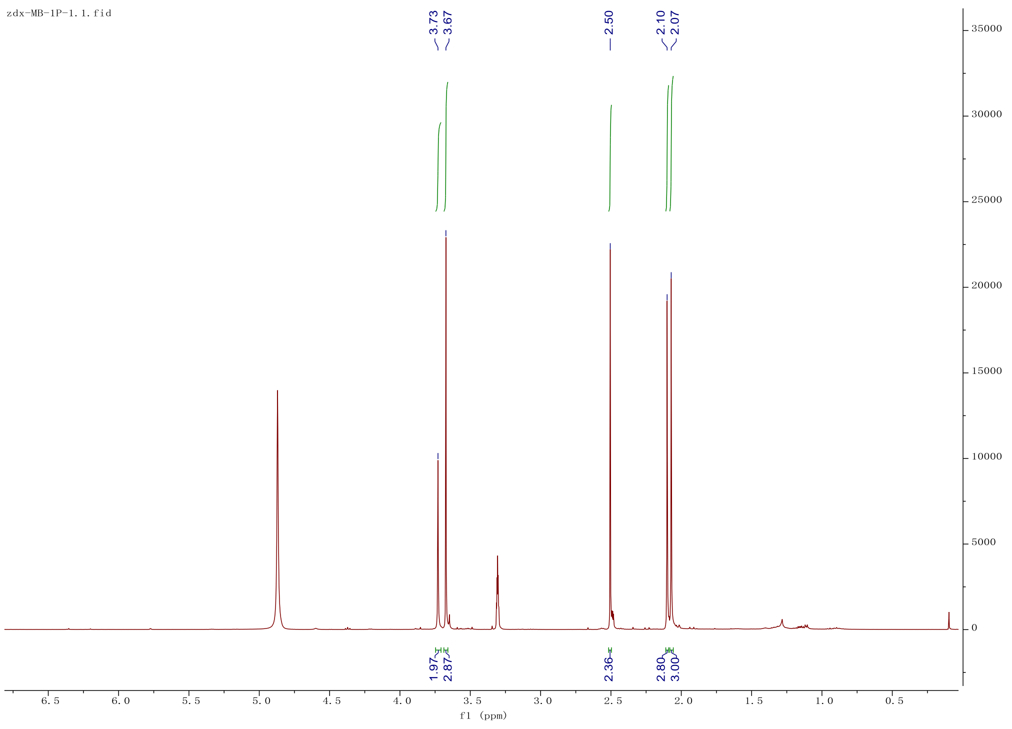

Figure S22. ^{1}H NMR (400 MHz, methanol-d_4) spectrum of compound **4**

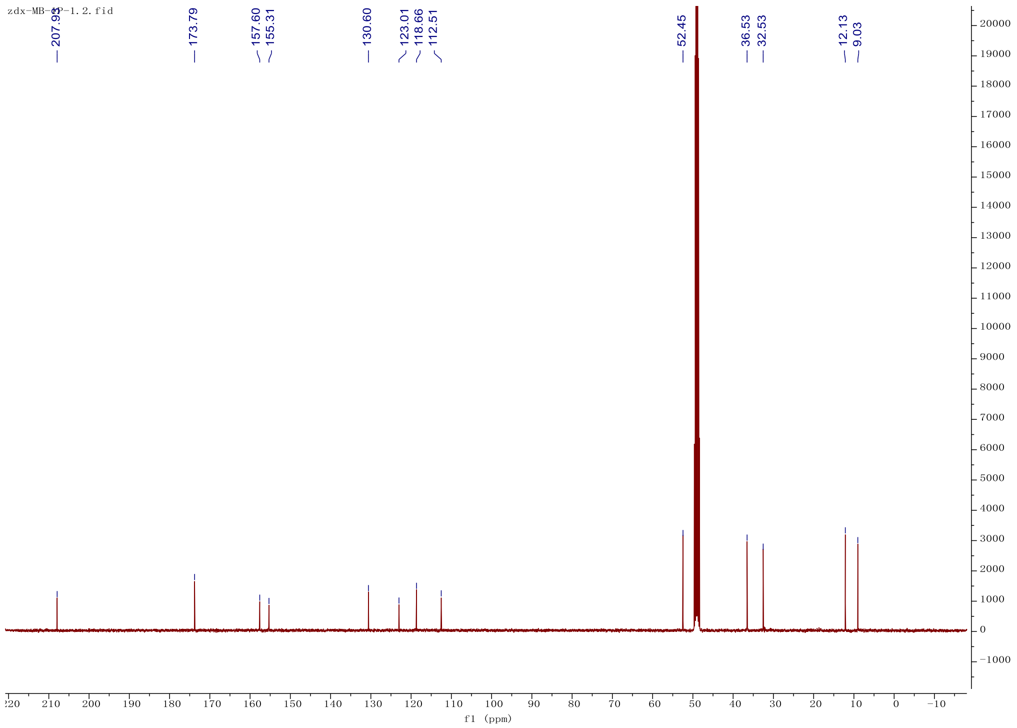

Figure S23. ^{13}C NMR (100 MHz, methanol-d_4) spectrum of compound **4**

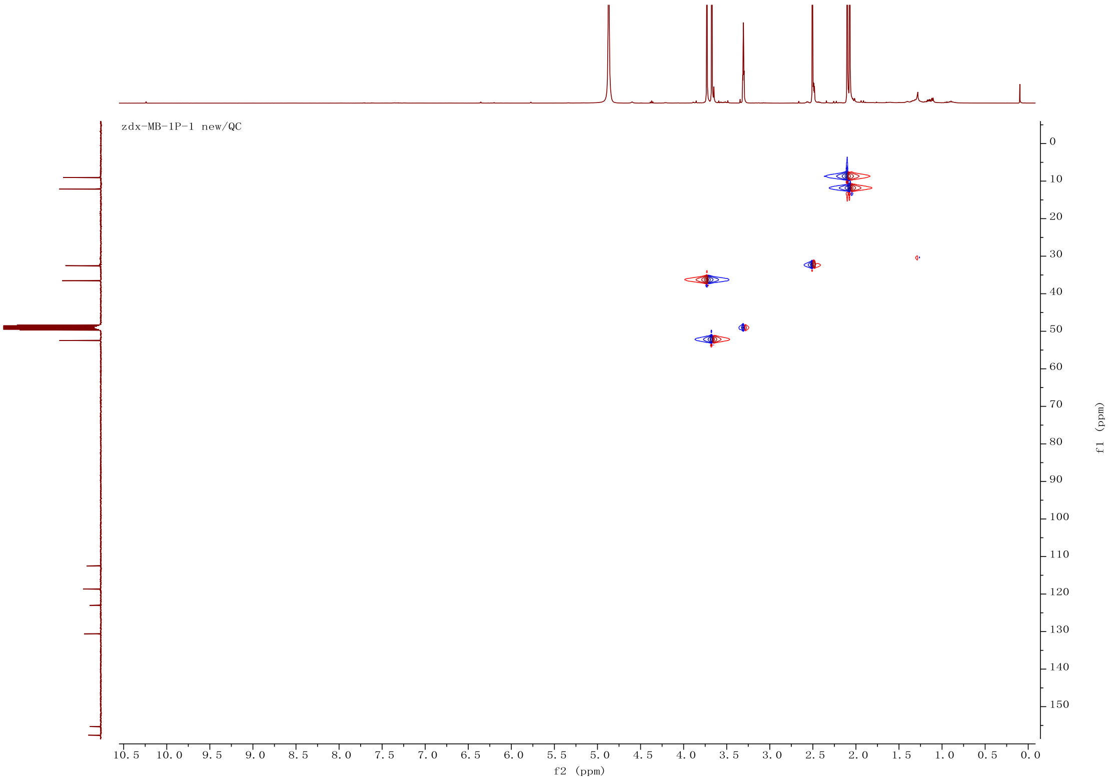

Figure S24. HMQC (methanol-d_4) spectrum of compound **4**

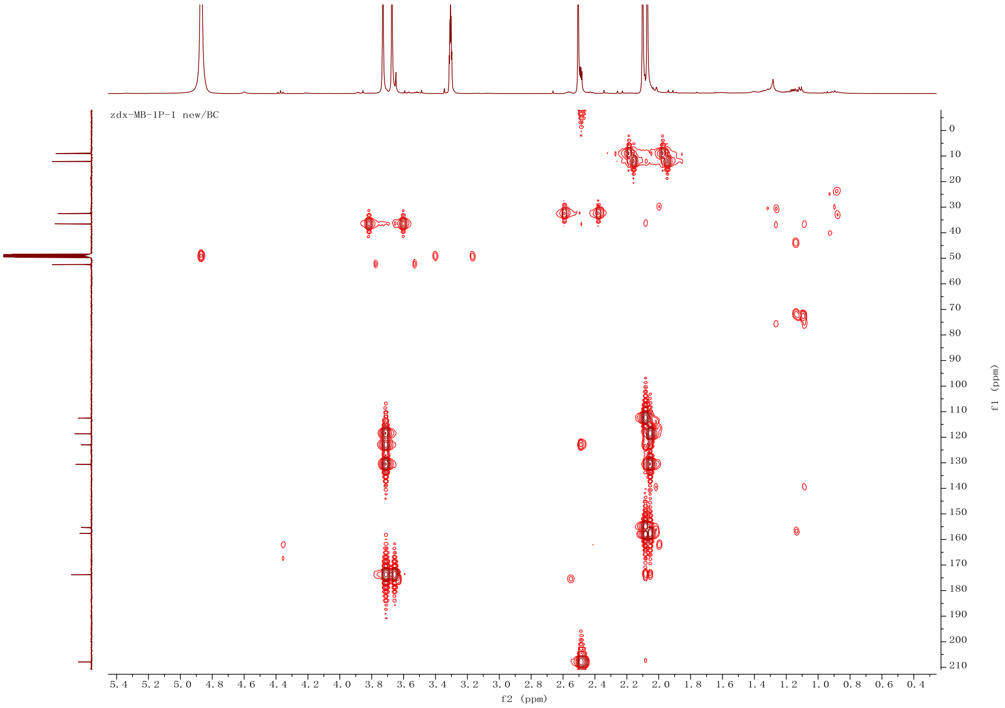

Figure S25. HMBC (methanol-d_4) spectrum of compound **4**

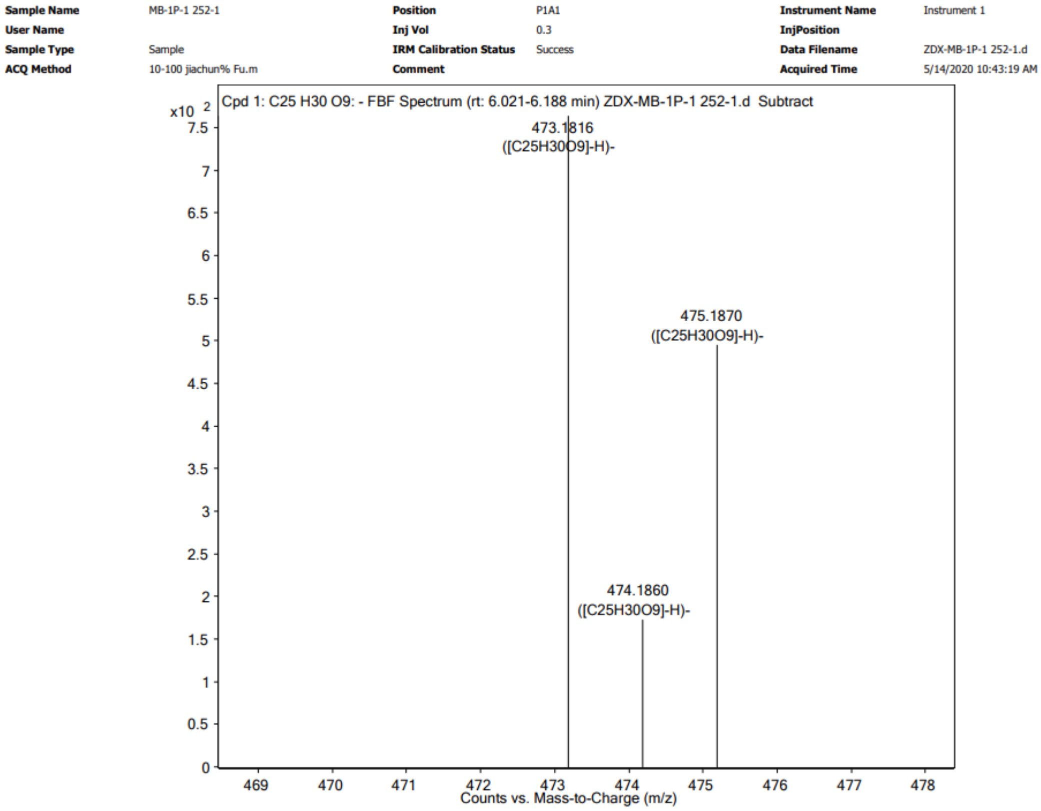

Figure S26. HR-ESI-MS spectrum of compound **4**

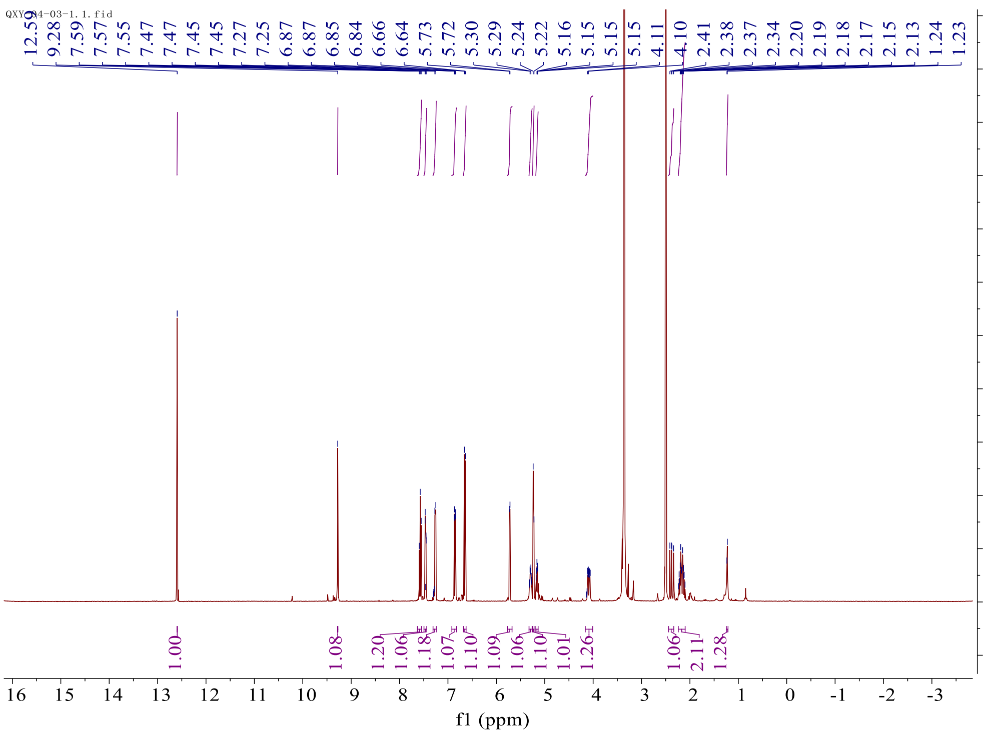

Figure S27. ^{1}H NMR (600 MHz, DMSO-d_6) spectrum of compound **5**

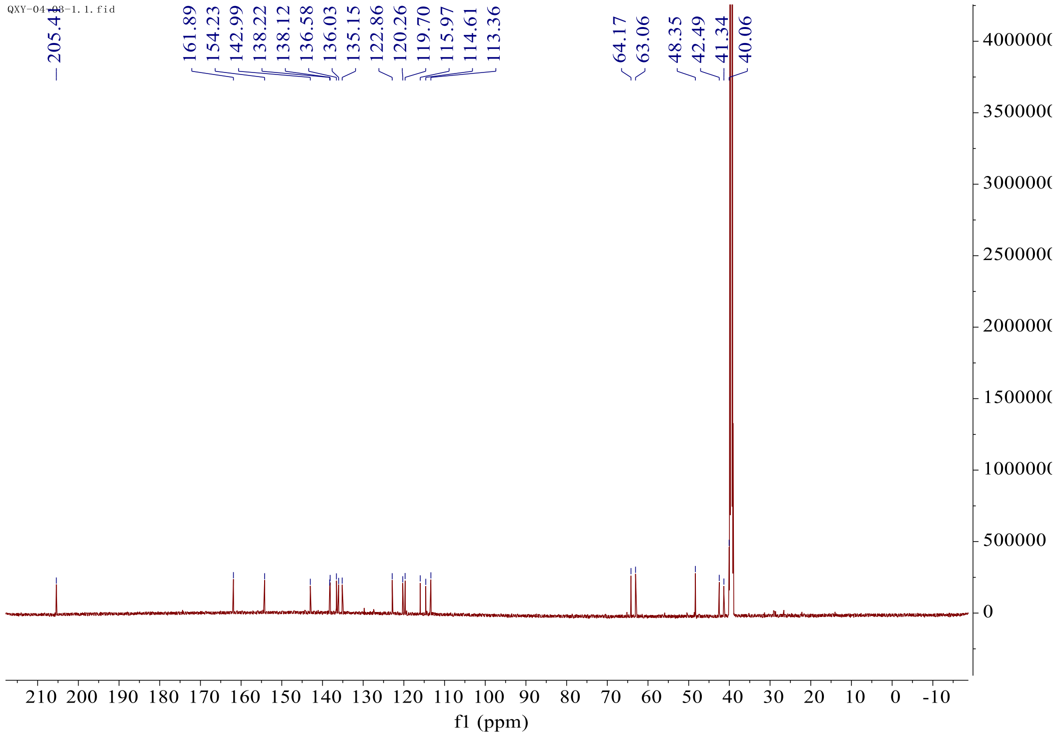

Figure S28. ^{13}C NMR (150 MHz, DMSO-d_6) spectrum of compound **5**

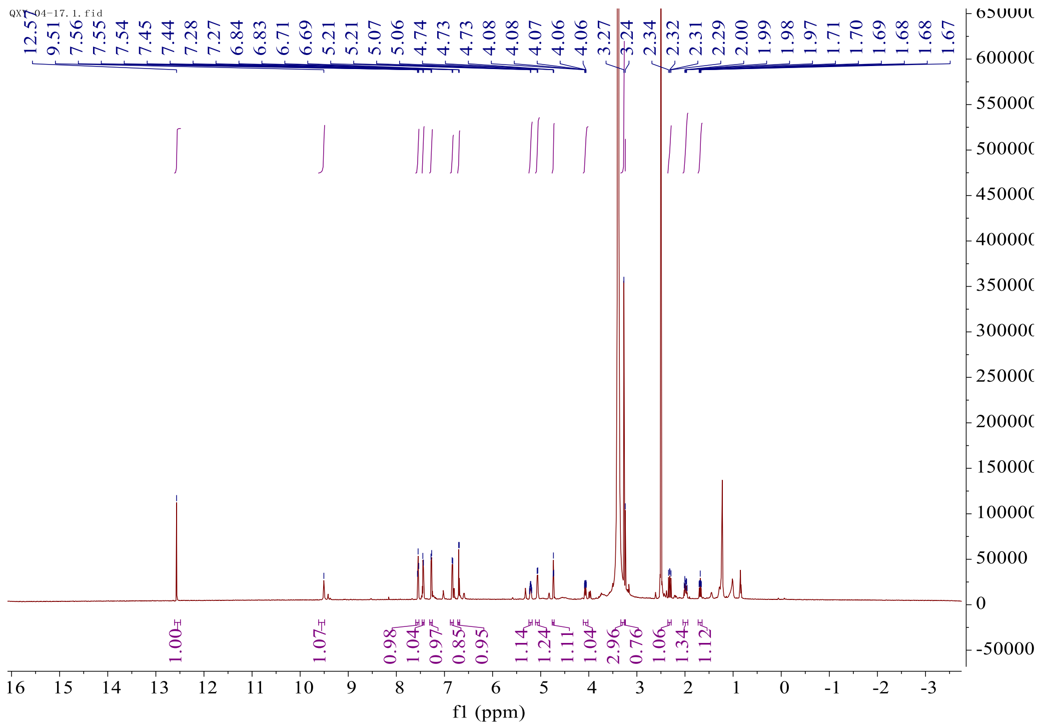

Figure S29. ^{1}H NMR (600 MHz, DMSO-d_6) spectrum of compound **6**

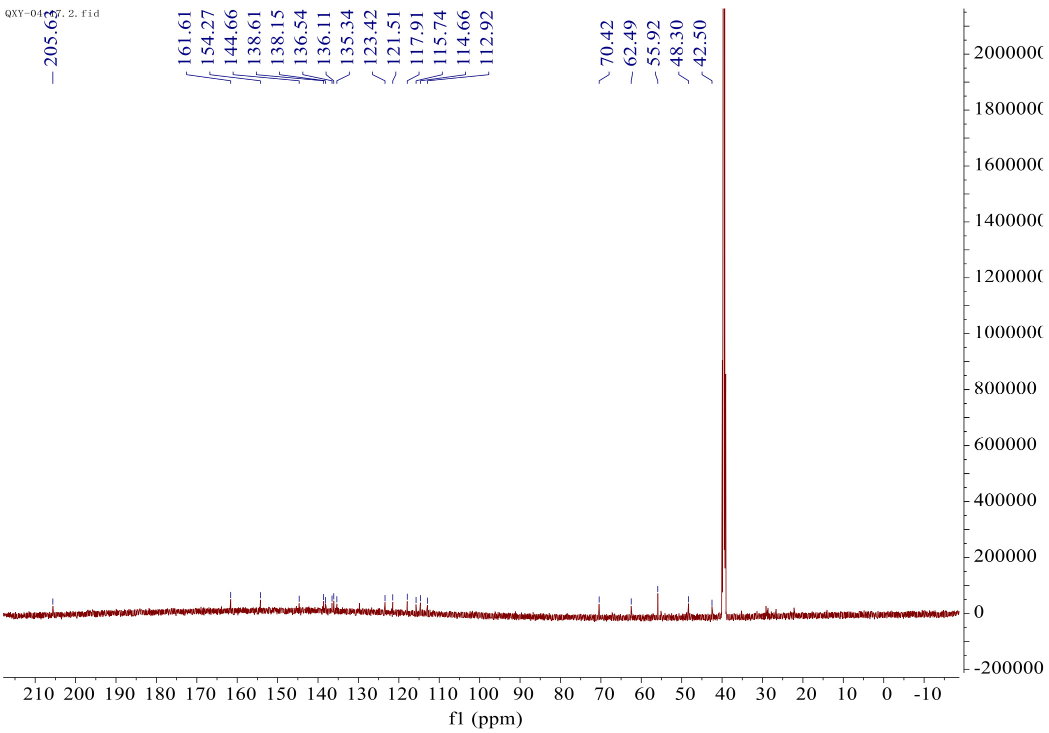

Figure S30. ^{13}C NMR (150 MHz, DMSO-d_6) spectrum of compound **6**

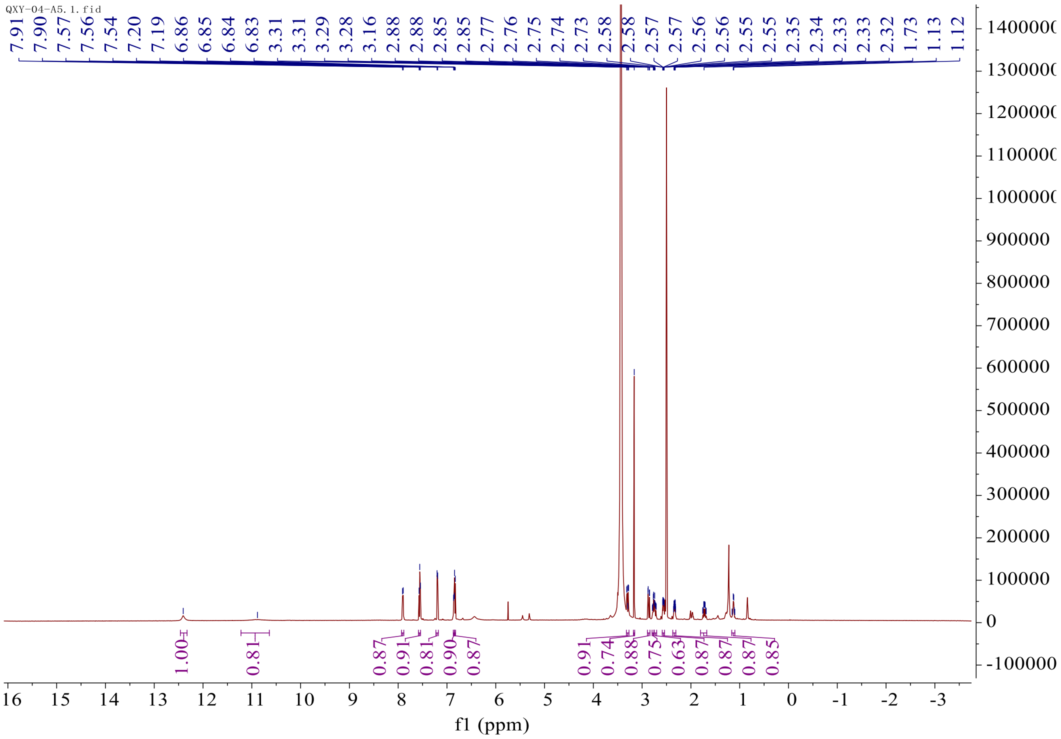

Figure S31. ^{1}H NMR (600 MHz, DMSO-d_6) spectrum of compound **7**

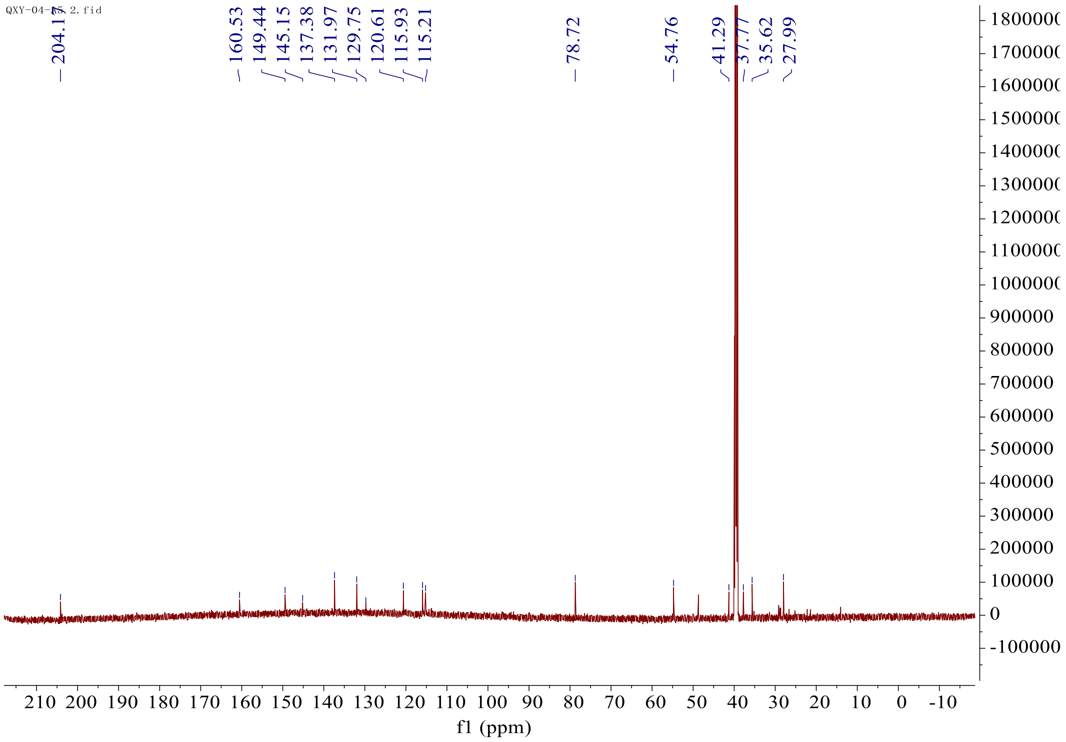

Figure S32. ^{13}C NMR (150 MHz, DMSO-d_6) spectrum of compound **7**

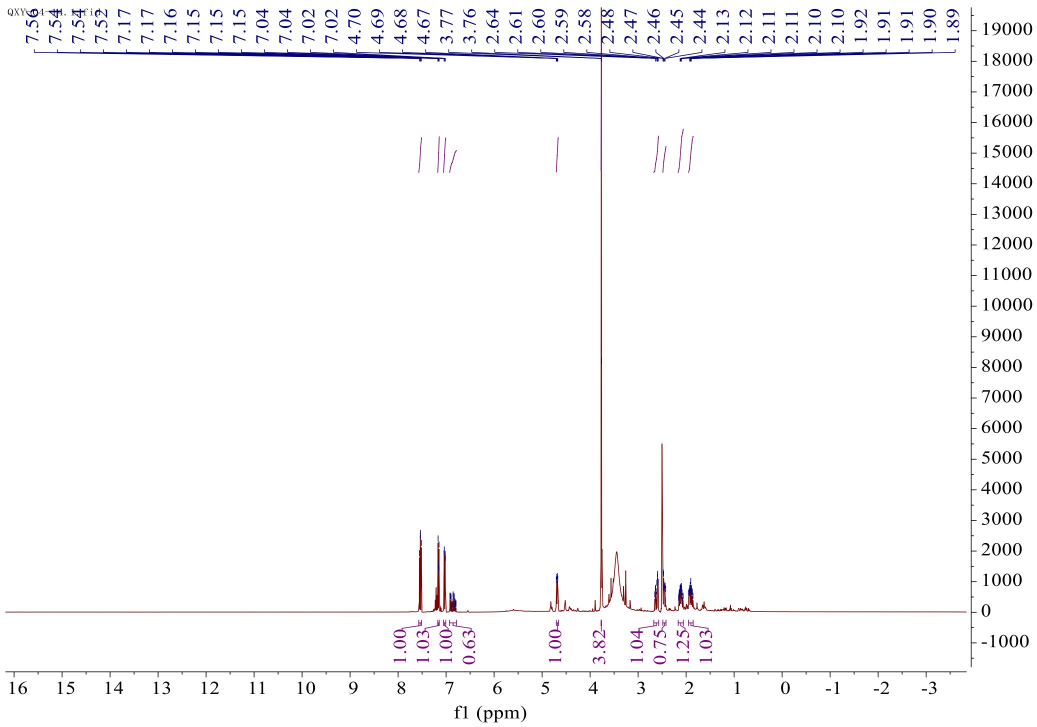

Figure S33. ^{1}H NMR (600 MHz, DMSO-d_6) spectrum of compound **8**

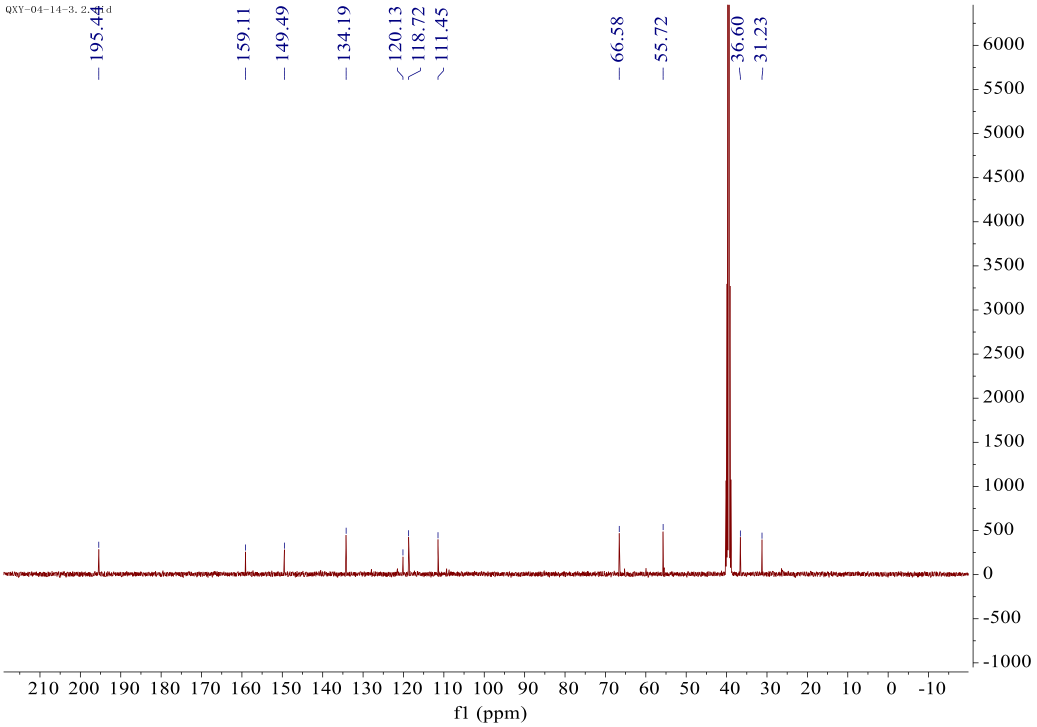

Figure S34. ^{13}C NMR (150 MHz, DMSO-d_6) spectrum of compound **8**

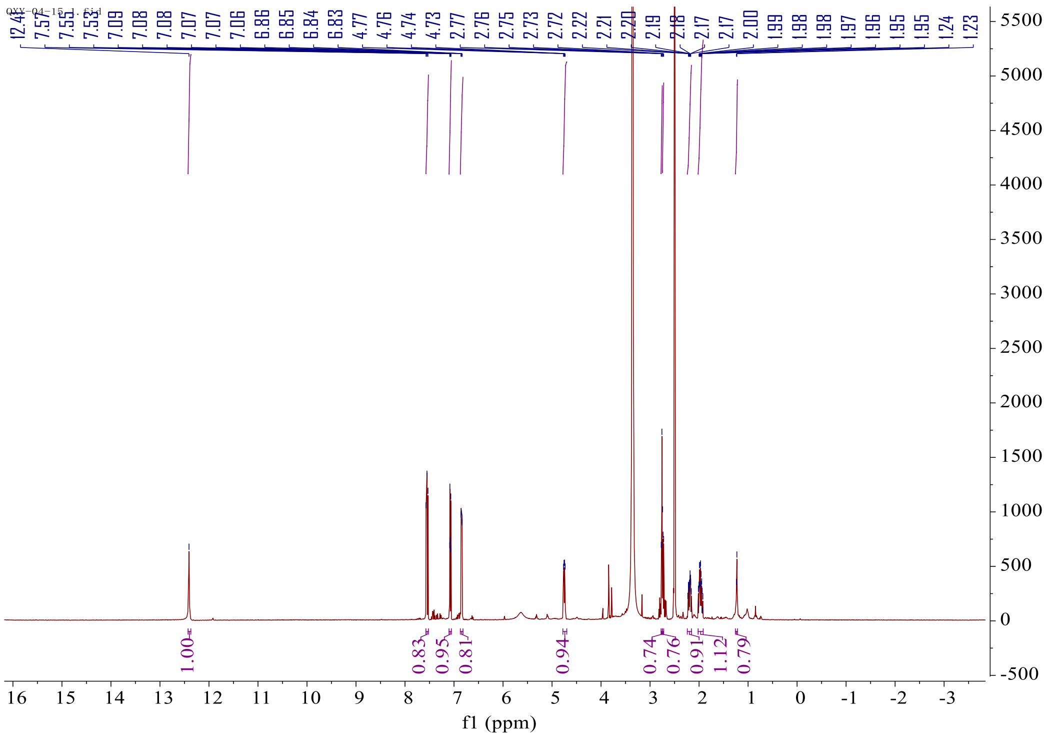

Figure S35. ^{1}H NMR (600 MHz, DMSO-d_6) spectrum of compound **9**

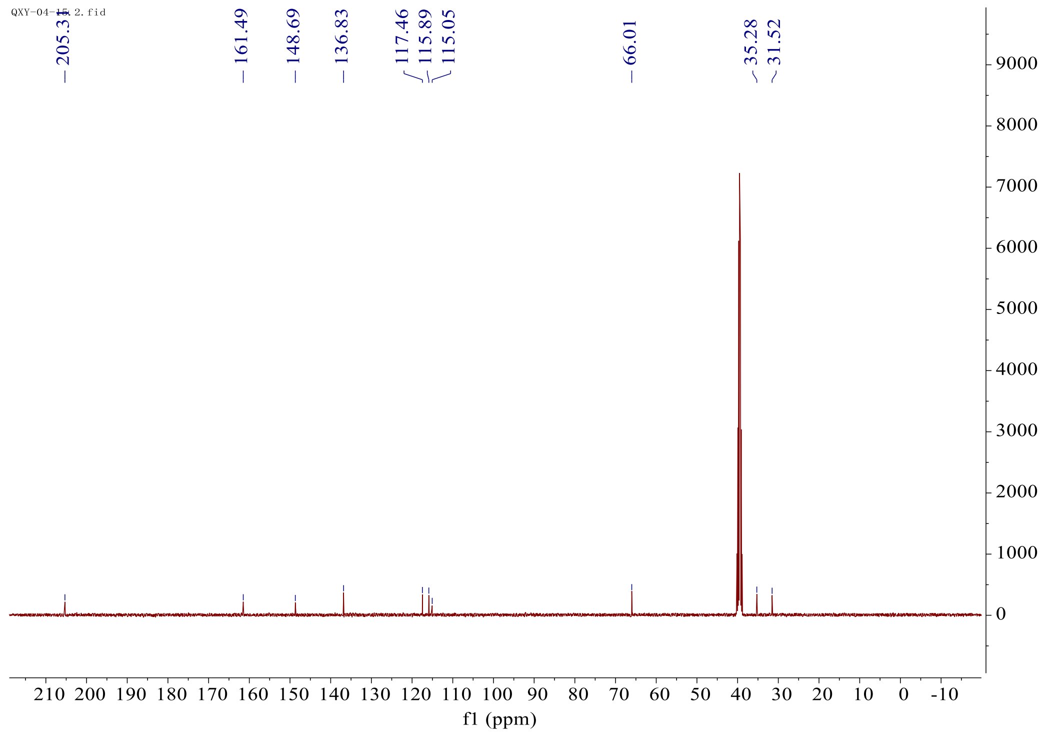

Figure S36. ^{13}C NMR (150 MHz, DMSO-d_6) spectrum of compound **9**

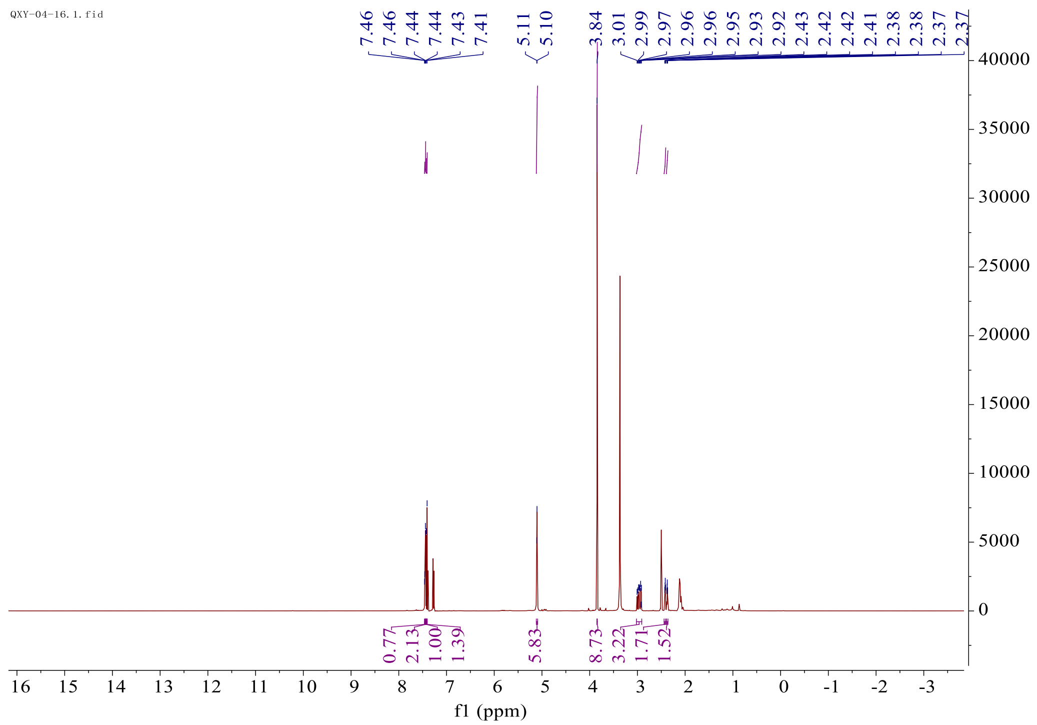

Figure S37. ^{1}H NMR (600 MHz, DMSO-d_6) spectrum of compound **10**

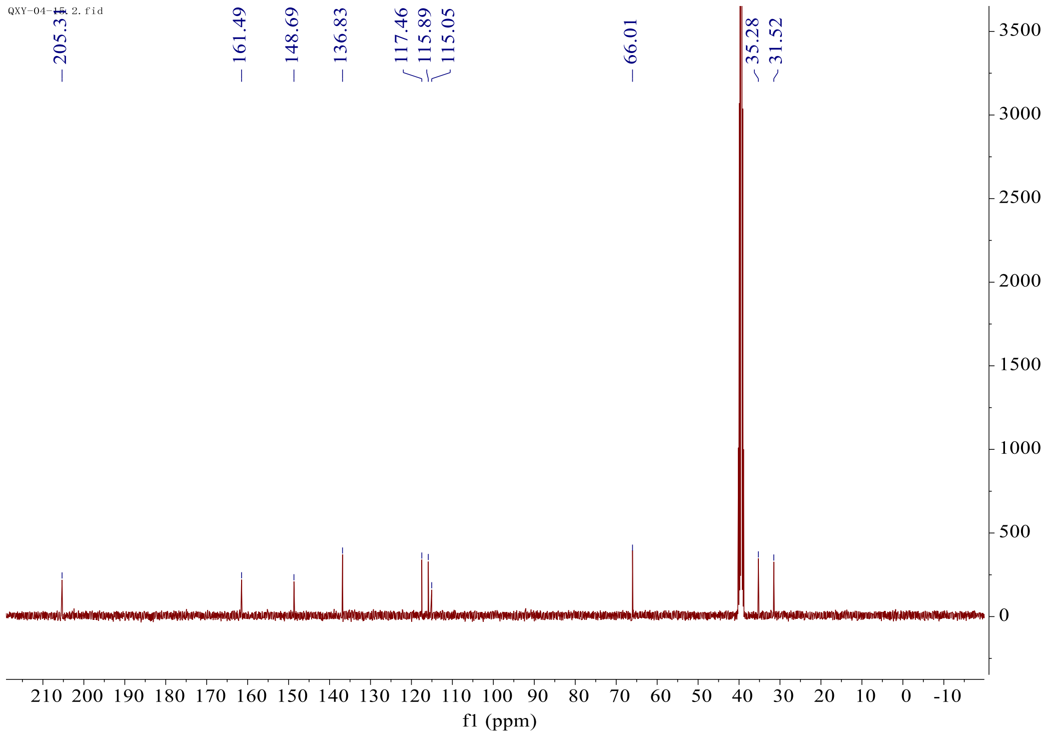

Figure S38. ^{13}C NMR (150 MHz, DMSO-d_6) spectrum of compound **10**

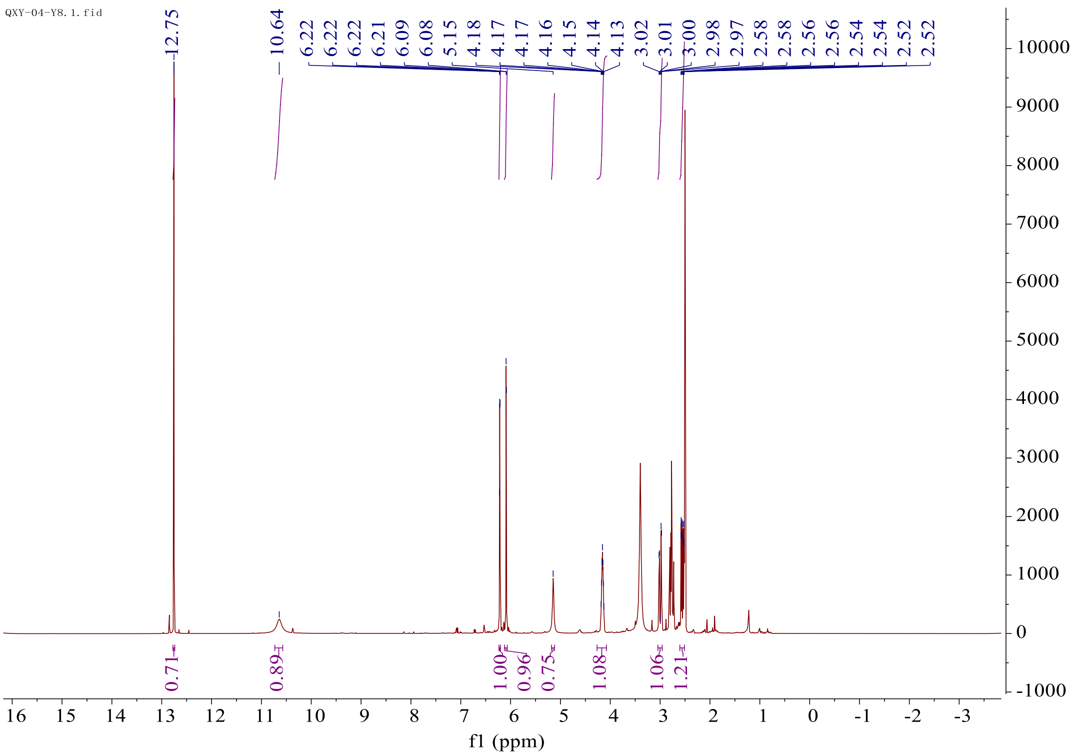

Figure S39. ^{1}H NMR (600 MHz, DMSO-d_6) spectrum of compound **11**

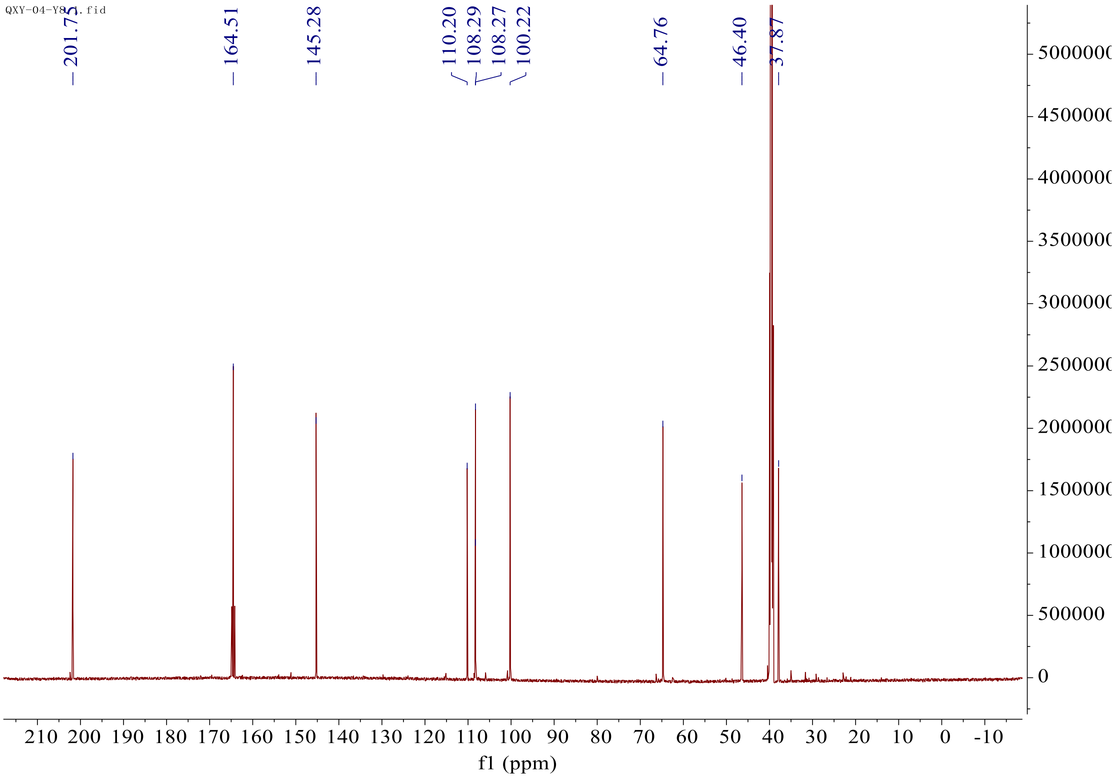

Figure S40. ^{13}C NMR (150 MHz, DMSO-d_6) spectrum of compound **11**

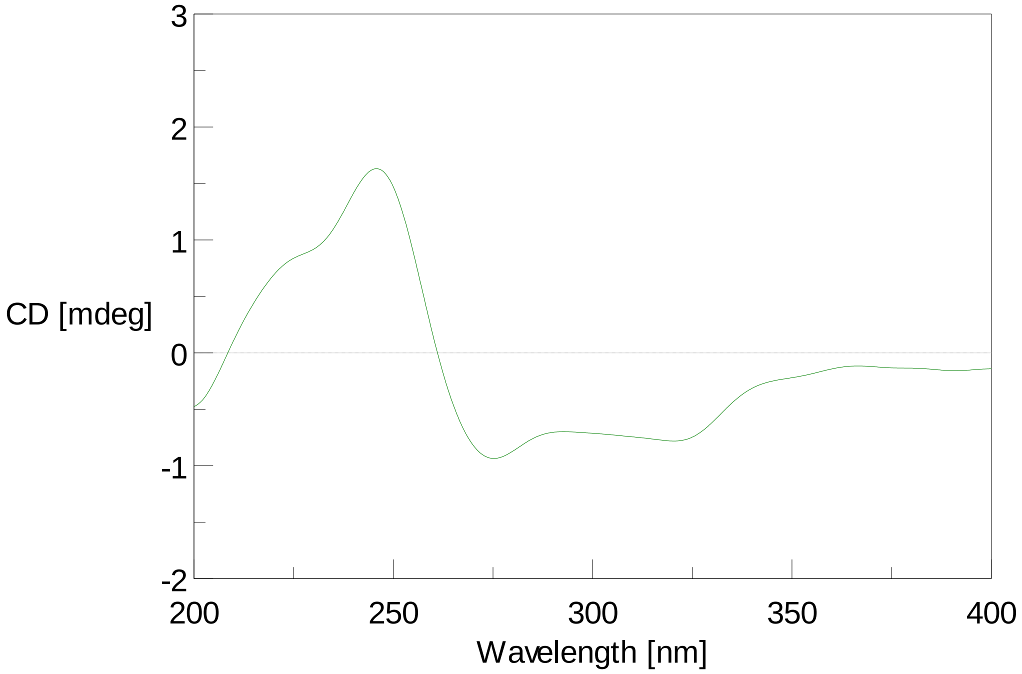

Figure S41. Experimental ECD of **1**

ECD calculation of **1**

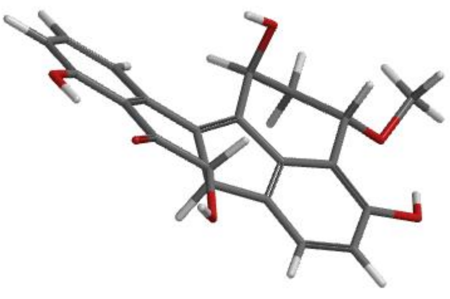

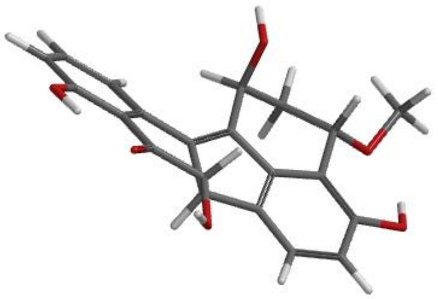

Conformer **1-1** 0 kJ/mol, 94.9%   Conformer **1-2** 8.97 kJ/mol, 2.6%

ECD calculation of **1** model

Table S1. Energy minimized coordinates of **1-1** model at the basis set def-SV(P) for all atoms(Å).

Atom	X	Y	Z

C	0	0	0
C	0	0	1.390507
C	1.206981	0	2.106643
C	2.435433	0.011046	1.414218
C	2.416908	-0.01618	0.012603
C	1.208333	-0.01532	-0.68895
C	1.182857	-0.01663	3.596701
C	2.496006	0.01787	4.375469
C	3.621834	-0.62	3.570399
C	3.686163	0.007639	2.176478
C	5.71358	0.358932	3.096434
C	5.00129	-0.31977	4.083963
C	5.596883	-0.61444	5.296634
C	6.924193	-0.21415	5.490024
C	7.623367	0.468978	4.481464
C	7.025117	0.780571	3.258038
C	7.644135	1.605848	2.153716
C	7.032059	1.23582	0.786095
C	5.496551	1.331406	0.785751
C	4.885922	0.569094	1.915646
O	-1.2326	-0.00189	1.98855
O	0.097144	-0.03538	4.18465
O	5.131145	2.70405	0.905549
O	9.064774	1.379563	2.14801
C	9.789609	2.437488	1.527738
O	8.922779	0.802111	4.744486
O	2.29727	-0.69339	5.602909
H	3.499348	-1.70794	3.492701
H	-0.94203	-0.00297	-0.54342
H	3.335583	-0.07343	-0.56278
H	1.209366	-0.03808	-1.77727
H	2.71017	1.064994	4.626463
H	5.058044	-1.14347	6.077043
H	7.424895	-0.43591	6.429234
H	7.445128	2.660298	2.388724
H	7.435844	1.901739	0.014898
H	7.343498	0.21897	0.508904
H	5.137821	0.978573	-0.18247
H	-1.1055	-0.0272	2.963725
H	4.162019	2.735951	0.981187
H	9.593596	3.387558	2.033783
H	10.85641	2.213161	1.614878
H	9.541972	2.5143	0.465986
H	9.328408	1.017305	3.8788

H	1.422129	-0.41498	35<br>5.939153

Table S2. Energy minimized coordinates of **1-2** model at the basis set def-SV(P) for all atoms(Å).

Atom	X	Y	Z
C	0	0	0
C	0	0	1.39036
C	1.208314	0	2.106162
C	2.434766	0.011195	1.4135
C	2.416841	-0.01482	0.012184
C	1.208516	-0.01438	-0.68966
C	1.185836	-0.02434	3.595559
C	2.500513	-0.00905	4.37358
C	3.619963	-0.64814	3.560288
C	3.6858	-0.00013	2.176754
C	5.709831	0.343152	3.10552
C	5.002476	-0.36237	4.077205
C	5.602122	-0.68786	5.279259
C	6.928059	-0.28925	5.480262
C	7.622909	0.424597	4.488822
C	7.018424	0.768136	3.277393
C	7.623915	1.635001	2.198468
C	7.014391	1.30417	0.818939
C	5.477582	1.376779	0.821701
C	4.879731	0.578043	1.93119
O	-1.23221	-0.00539	1.98848
O	0.099909	-0.03923	4.18555
O	5.074258	2.732929	0.992669
O	9.046687	1.426164	2.177066
C	9.759489	2.535733	1.637207
O	8.921259	0.75551	4.757317
O	2.295748	-0.73474	5.591916
H	3.490192	-1.73369	3.468109

H	-0.94136	-0.00223	-0.54395
H	3.337578	-0.06742	-0.5605
H	1.210372	-0.03475	-1.77704
H	2.723185	1.032424	4.636203
H	5.064715	-1.23631	6.047377
H	7.431323	-0.53486	6.41203
H	7.411593	2.678913	2.468305
H	7.409804	2.002028	0.071882
H	7.339465	0.302635	0.50753
H	5.112459	1.036988	-0.15029
H	-1.10189	-0.02087	2.963507
H	5.180696	3.177426	0.134761
H	9.572082	3.435864	2.229555
H	10.82813	2.307923	1.684495
H	9.489875	2.705184	0.591324
H	9.324048	0.994864	3.896949
H	1.425197	-0.44659	5.933152

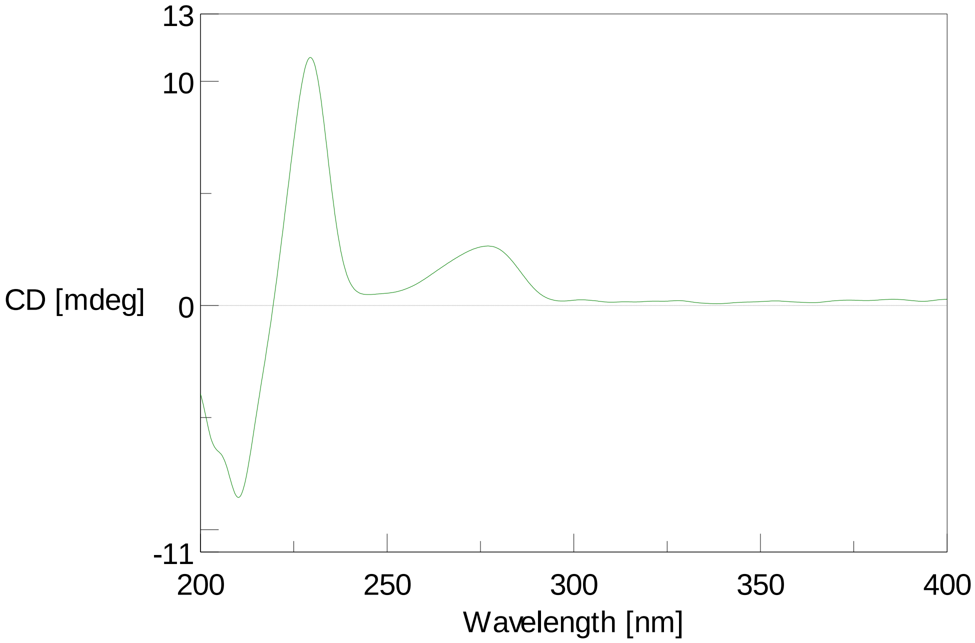

Figure S42. Experimental ECD of **2**

ECD calculation of **2**

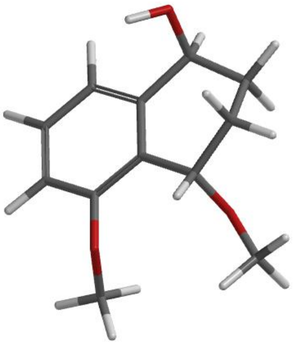

Conformer **2-1,** 0 kJ/mol, 32.9%

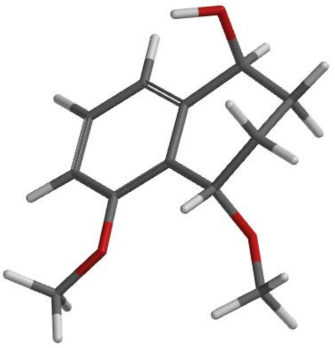

Conformer **2-2,** 0.22kJ/mol, 30.1%

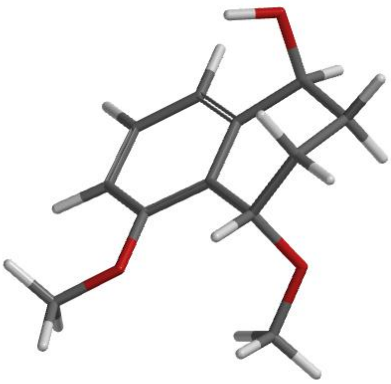

Conformer **2-3,** 0.88 kJ/mol, 23.1%

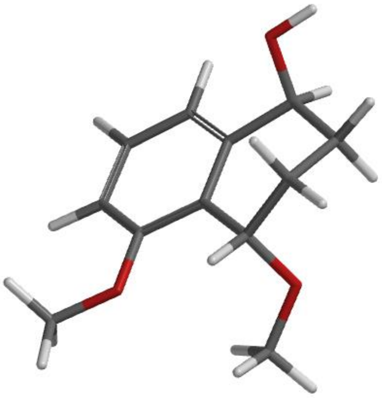

Conformer **2-4,** 4.94 kJ/mol, 4.6%

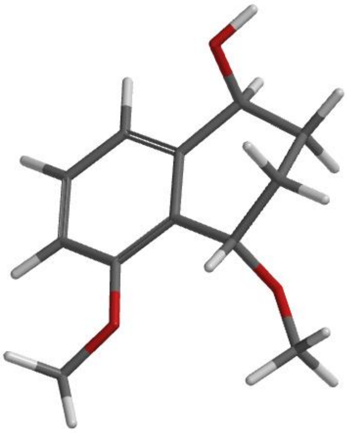

Conformer **2-5,** 5.32 kJ/mol, 3.8%

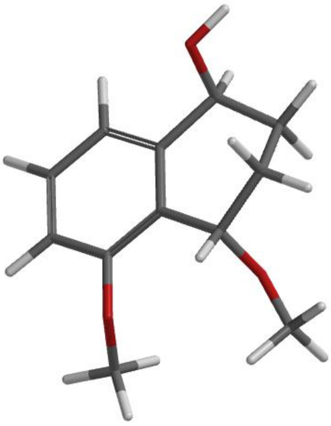

Conformer **2-5,** 7.64 kJ/mol, 3.4%

ECD calculation of **2** model

Table S3. Energy minimized coordinates of **2-1** model at the basis set def-SV(P) for all atoms(Å).

Atom	X	Y	Z

C	0	0	0
C	0	0	1.391134
C	1.21166	0	2.087532
C	2.441338	0.046246	1.40872
C	2.435458	0.041116	-0.00293
C	1.209451	0.017754	-0.6923
C	3.740393	0.049162	2.189915
C	4.931253	0.534886	1.354319
C	4.971907	-0.18899	0.009458
C	3.716925	0.124774	-0.8034
O	1.205523	0.025857	3.460316
C	0.73717	-1.21778	3.985219
O	3.853153	1.444629	-1.33844
O	3.981354	-1.30406	2.605414
C	4.600943	-1.37859	3.882407
H	-0.93977	-0.00491	-0.54444
H	-0.94314	0.008137	1.928573
H	1.197254	0.025369	-1.78166
H	3.63623	0.710816	3.059581
H	4.855274	1.617175	1.189872
H	5.871771	0.350395	1.883665
H	5.866586	0.125485	-0.541
H	5.050527	-1.26998	0.175066
H	3.668014	-0.56714	-1.65117
H	0.990386	-1.24611	5.049086
H	-0.3516	-1.29167	3.896328
H	1.220292	-2.07334	3.500251
H	3.122825	1.98599	-0.99305
H	3.945064	-0.95819	4.650864
H	4.775582	-2.43211	4.116794
H	5.56309	-0.85895	3.88148

Table S4. Energy minimized coordinates of **2-2** model at the basis set def-SV(P) for all atoms(Å).

Atom	X	Y	Z
C	0	0	0
C	0	0	1.393617
C	1.206343	0	2.102885
C	2.433797	0.015947	1.404252
C	2.427887	0.014935	-0.00959
C	1.203981	0.009319	-0.69718
C	3.743061	-0.02406	2.171678
C	4.93728	0.457668	1.339905

C	4.963927	-0.24974	-0.01287
C	3.708269	0.084126	-0.81461
O	1.290466	0.017797	3.47212
C	0.111507	-0.31227	4.196967
O	3.859132	1.405759	-1.34368
O	3.971382	-1.39013	2.545205
C	4.484326	-1.51499	3.86347
H	-0.94156	-0.00275	-0.54378
H	-0.95895	0.006519	1.8992
H	1.189187	0.017305	-1.78576
H	3.666611	0.621063	3.057717
H	4.877677	1.543164	1.190791
H	5.874386	0.249808	1.867386
H	5.859297	0.062391	-0.56471
H	5.03328	-1.33361	0.138756
H	3.645489	-0.60125	-1.66754
H	-0.3026	-1.2733	3.872982
H	0.385233	-0.4071	5.252603
H	-0.63156	0.487626	4.117662
H	3.145506	1.95727	-0.98119
H	3.757524	-1.14615	4.593703
H	4.668386	-2.57403	4.061817
H	5.429272	-0.9716	3.965129

Table S5. Energy minimized coordinates of **2-3** model at the basis set def-SV(P) for all atoms(Å).

Atom	X	Y	Z
C	0	0	0
C	0	0	1.393825
C	1.205227	0	2.103934
C	2.435028	-0.01129	1.406554
C	2.428853	0.025852	-0.0077
C	1.203188	0.022079	-0.69509
C	3.753763	-0.08567	2.161615
C	4.87597	0.595292	1.365576
C	4.991086	-0.02868	-0.02629
C	3.700637	0.167435	-0.81782
O	1.289375	0.026498	3.472921
C	0.07338	0.017771	4.210209
O	3.739574	1.483291	-1.3847
O	4.167569	-1.45012	2.340137
C	3.545346	-2.10565	3.434378
H	-0.9415	-0.0002	-0.5441

H	-0.96111	0.000969	1.895862
H	1.190411	0.054802	-1.78358
H	3.694068	0.423175	3.132226
H	4.68994	1.672721	1.281975
H	5.831399	0.462942	1.887786
H	5.837797	0.423986	-0.55644
H	5.206514	-1.10034	0.06773
H	3.689386	-0.54261	-1.65272
H	-0.49986	-0.89364	4.015203
H	0.328674	0.027799	5.274392
H	-0.51939	0.914925	4.002788
H	3.102607	2.034495	-0.89972
H	2.500269	-2.33162	3.208065
H	4.061991	-3.05543	3.596532
H	3.624412	-1.51167	4.349925

Table S6. Energy minimized coordinates of **2-4** model at the basis set def-SV(P) for all atoms(Å).

Atom	X	Y	Z
C	0	0	0
C	0	0	1.39313
C	1.205978	0	2.102889
C	2.435499	-0.02052	1.404328
C	2.430791	0.007941	-0.01006
C	1.204708	0.013161	-0.69483
C	3.753876	-0.0772	2.160646
C	4.870041	0.601671	1.354305
C	4.996635	-0.05009	-0.02141
C	3.704566	0.108272	-0.82166
O	1.290087	0.028658	3.472386
C	0.075286	0.113203	4.205429
O	3.705699	1.399636	-1.43843
O	4.181259	-1.43596	2.35882
C	3.546774	-2.08752	3.448358
H	-0.94036	0.009923	-0.54472
H	-0.96157	0.0068	1.894288
H	1.191835	0.053778	-1.78316
H	3.690834	0.4439	3.12405
H	4.671519	1.675437	1.248033
H	5.824933	0.491655	1.88185
H	5.841862	0.396852	-0.55824
H	5.224115	-1.11679	0.096755
H	3.684738	-0.63959	-1.62303

H	-0.54339	-0.77553	4.046995
H	0.330433	0.154009	5.268868
H	-0.47223	1.029071	3.960701
H	4.481479	1.435438	-2.02064
H	4.070798	-3.03063	3.628502
H	3.604771	-1.48494	4.358468
H	2.50763	-2.32697	3.206124

Table S7. Energy minimized coordinates of **2-5** model at the basis set def-SV(P) for all atoms(Å).

Atom	X	Y	Z
C	0	0	0
C	0	0	1.39441
C	1.20638	0	2.102692
C	2.433637	0.006675	1.402545
C	2.429633	-0.00343	-0.01184
C	1.204067	0.001517	-0.69619
C	3.743189	-0.01291	2.169901
C	4.930803	0.467335	1.328425
C	4.968252	-0.27278	-0.00654
C	3.707534	0.024735	-0.81792
O	1.294148	0.018389	3.472861
C	0.094291	-0.19113	4.20518
O	3.818314	1.327104	-1.39306
O	3.991046	-1.37179	2.559692
C	4.459661	-1.47381	3.896653
H	-0.94087	0.009713	-0.54351
H	-0.95943	0.011256	1.898569
H	1.190864	0.021495	-1.78428
H	3.663477	0.643951	3.045597
H	4.857595	1.548233	1.152733
H	5.870016	0.283027	1.861599
H	5.861753	0.033493	-0.56362
H	5.048835	-1.35132	0.171988
H	3.63641	-0.69791	-1.64078
H	-0.59311	0.652971	4.082023
H	-0.3835	-1.13703	3.928706
H	0.357367	-0.25093	5.26623
H	4.59521	1.316464	-1.9746
H	3.698584	-1.1172	4.597422
H	4.660616	-2.52611	4.111282
H	5.387085	-0.90756	4.026049

Table S8. Energy minimized coordinates of **2-6** model at the basis set def-SV(P) for all atoms(Å).

Atom	X	Y	Z
C	0	0	0
C	0	0	1.390728
C	1.21056	0	2.08642
C	2.440954	0.041706	1.40626
C	2.436806	0.025412	-0.00599
C	1.209045	0.009848	-0.69226
C	3.738965	0.069619	2.186019
C	4.924248	0.550821	1.34054
C	4.975017	-0.20878	0.016524
C	3.716679	0.066797	-0.80737
O	1.206518	0.0297	3.459299
C	0.72762	-1.20728	3.987844
O	3.81354	1.367172	-1.38997
O	3.998725	-1.27283	2.626752
C	4.588722	-1.31451	3.91871
H	-0.9395	0.006017	-0.54583
H	-0.94346	0.016445	1.928087
H	1.198654	0.02862	-1.78131
H	3.629801	0.747753	3.042915
H	4.835279	1.627561	1.14717
H	5.86594	0.390766	1.876745
H	5.869212	0.097004	-0.53953
H	5.063956	-1.28405	0.21185
H	3.660215	-0.66136	-1.62444
H	0.980196	-1.23496	5.051984
H	-0.36107	-1.27417	3.899681
H	1.204925	-2.0685	3.505106
H	4.592712	1.364205	-1.96859
H	3.905917	-0.89796	4.665063
H	4.783016	-2.35926	4.17238
H	5.538422	-0.77057	3.931473

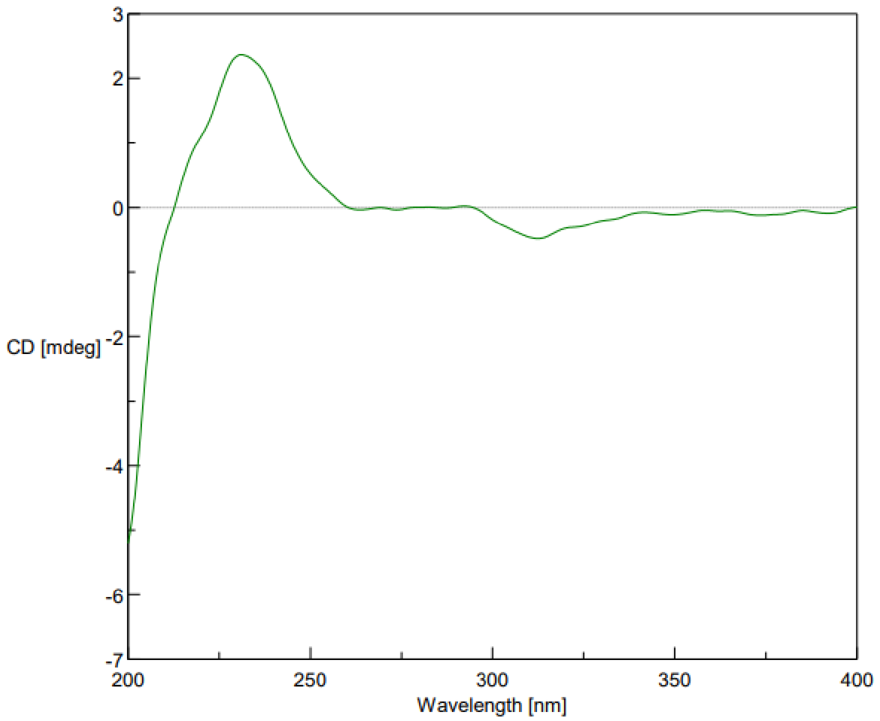

Figure S43. Experimental ECD of **3**

ECD calculation of **3**

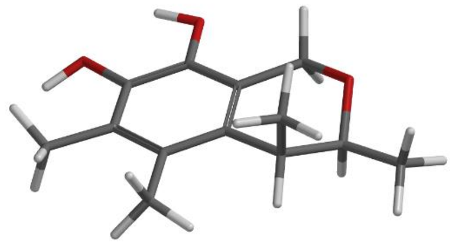

Conformer **3-1** 0 kJ/mol, 96.9%

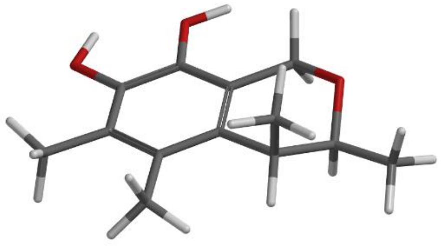

Conformer **3-2** 8.94 kJ/mol, 2.6%

ECD calculation of **3** model

Table S9. Energy minimized coordinates of **3-1** model at the basis set def-SV(P) for all atoms(Å).

Atom	X	Y	Z

C	0	0	0
C	0	0	1.424333
C	1.238133	0	2.134091
C	2.461205	-0.02906	1.424601
C	2.429986	-0.09551	0.035242
C	1.226265	-0.09308	-0.66766
O	1.342512	-0.19382	-2.03268
O	3.601455	-0.17519	-0.6721
C	-1.28973	0.067066	-0.77867
C	3.797712	-0.05007	2.129669
C	-1.32384	0.053396	2.154767
C	1.315216	0.074584	3.659723
C	2.661602	-0.51232	4.144461
C	1.1032	1.5289	4.104947
O	3.731777	0.211621	3.536074
C	2.849434	-0.43819	5.655081
H	0.481479	-0.4538	-2.39748
H	3.317656	-0.18026	-1.60806
H	-2.06059	0.634351	-0.24891
H	-1.15432	0.591063	-1.73098
H	-1.66026	-0.94336	-0.97621
H	4.458088	0.713013	1.702868
H	4.269869	-1.02996	1.990654
H	-1.72137	1.072926	2.127533
H	-2.04508	-0.63076	1.694994
H	-1.25844	-0.2581	3.197574
H	0.536002	-0.55539	4.099502
H	2.734939	-1.57006	3.853551
H	1.058296	1.605264	5.19569
H	0.161947	1.934453	3.71994
H	1.907282	2.182873	3.751101
H	2.964376	0.593784	6.001248
H	3.771491	-0.95708	5.941299
H	2.013617	-0.90598	6.184707

Table S10. Energy minimized coordinates of **3-2** model at the basis set def-SV(P) for all atoms(Å).

Atom	X	Y	Z

C	0	0	0
C	0	0	1.418143
C	1.240864	0	2.123133
C	2.471651	-0.0303	1.412336
C	2.437068	-0.0793	0.016179
C	1.222024	-0.06406	-0.66966
O	1.254905	-0.11574	-2.04008
O	3.556046	-0.14609	-0.7783
C	-1.28397	0.027769	-0.78949
C	3.790475	-0.06689	2.152467
C	-1.32642	0.063896	2.141828
C	1.303903	0.071377	3.650708
C	2.631831	-0.53903	4.147956
C	1.109318	1.527312	4.096121
O	3.71702	0.174529	3.555867
C	2.803085	-0.47834	5.661376
H	2.207662	-0.15388	-2.25801
H	4.356909	-0.10674	-0.23402
H	-1.97782	0.769377	-0.38013
H	-1.1242	0.309902	-1.83538
H	-1.75435	-0.96078	-0.77249
H	4.459762	0.700927	1.748175
H	4.262375	-1.04508	2.001595
H	-1.68929	1.096941	2.157927
H	-2.07249	-0.56876	1.650462
H	-1.27773	-0.29975	3.16854
H	0.509719	-0.54683	4.083642
H	2.694533	-1.59581	3.850045
H	1.057029	1.603848	5.186036
H	0.177637	1.947186	3.703818
H	1.926303	2.17021	3.748904
H	2.927509	0.549072	6.016053
H	3.714889	-1.01104	5.954041
H	1.954816	-0.93758	6.17757
